# A Chemically Soldered Polyoxometalate Single‐Molecule Transistor†

**DOI:** 10.1002/anie.202002174

**Published:** 2020-05-12

**Authors:** Chuanli Wu, Xiaohang Qiao, Craig M. Robertson, Simon J. Higgins, Chenxin Cai, Richard J. Nichols, Andrea Vezzoli

**Affiliations:** ^1^ Department of Chemistry University of Liverpool Crown Street Liverpool L69 7ZD UK; ^2^ School of Chemistry and Materials Science Nanjing Normal University Nanjing 210023 P. R. China; ^3^ Stephenson Institute for Renewable Energy University of Liverpool Peach Street Liverpool L69 7ZF UK

**Keywords:** charge transfer, electrochemical transistor, molecular devices, molecular electronics, polyoxometalates

## Abstract

Polyoxometalates have been proposed in the literature as nanoelectronic components, where they could offer key advantages with their structural versatility and rich electrochemistry. Apart from a few studies on their ensemble behaviour (as monolayers or thin films), this potential remains largely unexplored. We synthesised a pyridyl‐capped Anderson–Evans polyoxometalate and used it to fabricate single‐molecule junctions, using the organic termini to chemically “solder” a single cluster to two nanoelectrodes. Operating the device in an electrochemical environment allowed us to probe charge transport through different oxidation states of the polyoxometalate, and we report here an efficient three‐state transistor behaviour. Conductance data fits a quantum tunnelling mechanism with different charge‐transport probabilities through different charge states. Our results show the promise of polyoxometalates in nanoelectronics and give an insight on their single‐entity electrochemical behaviour.

## Graphical abstract

**An Anderson–Evans polyoxometalate** functionalised with 4‐pyridyl termini has been used to fabricate single‐entity junctions in an electrochemical environment. As the charge state of the polyoxomolybdate is changed by the electrochemical potential, the fabricated devices show a tunnelling transistor behaviour, with OFF‐ON‐OFF conductance gating by more than one order of magnitude.

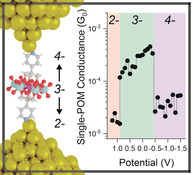

## Introduction

Assemblies of molecules, or even single molecules, might offer new approaches to traditional nanoelectronics and nanofabrication in the future, and contribute to scalability with a bottom‐up self‐assembly approach and miniaturisation.^1^ A key aspect of molecular electronics from the perspective of chemists is, however, the possibility of exploring a large chemical space to i) tune the device response to the desired range and magnitude, obtaining, in turn, important insights on structure–property relationships, and ii) study phenomena unique to the molecular and quantum world. Since the first pioneering studies in the late 1990s,^2^ single‐molecule devices with behaviour like semiconductor‐based diodes,^3^, ^4^ resistors,^5^ switches,^6^, ^7^, ^8^, ^9^ and transistors^10^, ^11^, ^12^, ^13^ have been demonstrated, and the chemical complexity of the molecules used to fabricate junctions has rapidly increased. Stemming from the original studies employing simple aliphatic and conjugated rod‐like oligoaryl moieties,^14^ it is now common to read reports on molecular wires incorporating fused polyaromatic/heterocyclic systems,^15^, ^16^ supramolecular complexes,^17^, ^18^ organometallic centres,^19^, ^20^ and, more recently, polynuclear clusters.^21^, ^22^, ^23^, ^24^ The latter are particularly interesting from a technology point of view for their electronic behaviour, as the presence of multiple metallic centres imparts stability to several oxidation states, with the cluster accommodating large charge variations. These electron‐sink phenomena^25^, ^26^ (that is, clusters are able to accept and release electrons reversibly without significant changes in their structure) resulted in great interest in their synthesis and applications, especially as electroactive materials to be deployed in electronic, sensing, and catalytic devices.^27^, ^28^

**Figure 1:**
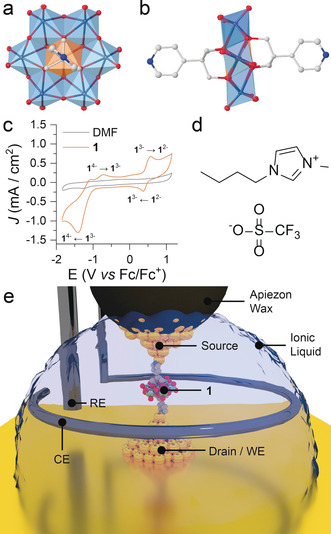
Details on the experiments presented in this study: a) top view of **1** (SCXRD structure). b) Side view of **1** (SCXRD structure). See the Supporting Information for more details on the structure of **1**. c) Cyclic voltammetry of **1** (10 mm) in DMF with 0.1 m NBu_4_BF_4_ as the support electrolyte using a glassy‐carbon working electrode, a Pt‐wire counter electrode, and a Pt‐wire quasi‐reference electrode. Ferrocene was added at the end of the experiment as a standard (see the Supporting Information for calibration and additional data). Scan speed 100 mV s^−1^. d) Structure of the ionic liquid BMIM–OTf used in this study. e) Depiction of the single‐POM junction in the four‐electrode configuration controlled by the bipotentiostat. WE=working electrode; CE=counter electrode; RE=reference electrode. Legend for panels (a) and (b): teal polyhedra: {MoO_6_}; orange polyhedra: {MnO_6_}; grey spheres: C; blue spheres: N; red ellipsoids: O. H atoms and counterions (3 NBu_4_^+^) not shown for clarity.

Polyoxometalates (POMs) are a class of cluster compounds noteworthy for their stability and rich electrochemistry.^29^ The relatively high oxidation state of the metallic centres linked together by μ_2_‐oxido ligands gives them less susceptibility to decomposition both in ambient atmosphere and in solution, resulting in stability over multiple redox states. In recent years, much effort has been devoted to the functionalisation of POMs with organic moieties,^30^ to impart properties such as biocompatibility^31^ and luminescence,^32^, ^33^ and to provide additional metal‐binding sites for the synthesis of coordination polymers and three‐dimensional frameworks.^34^ Metallophilic termini can, however, also be exploited in molecular electronics as electrode‐anchoring groups,^35^, ^36^ to ensure clear junction formation and provide strong mechanical stability and high electronic coupling between the molecule and the source/drain electrodes. While perspectives on the promise of polyoxometalates as molecular electronic components have indeed been featured in publications,^37^, ^38^ experimental reports are limited to a few niche studies, mostly on their behaviour as a nanoscale ensemble (that is, a monolayer or a thin film)^22^, ^39^, ^40^ or immobilised on a metal surface.^41^ We therefore focussed on the synthesis of the Anderson–Evans cluster (Figure 1 a,b) functionalised with pyridyl ligands, **1**, (NC_5_H_4_)−C−(CH_2_O)_3_≡[MnMo_6_O_18_]≡(OCH_2_)_3_−C−(C_5_H_4_N), to then use it as molecular wire for the fabrication of chemically soldered^21^ single‐polyoxometalate junctions. In this contribution, we explore its single‐entity electrochemistry, and we find a remarkable three‐state transistor behaviour, with both ON/OFF ratios exceeding one order of magnitude.

## Results and Discussion

We followed a published procedure^34^ for the synthesis of compound **1** (more details are given in the Supporting Information) as a tris(tetrabutylammonium) salt. To confirm the structure of the polyoxometalate, we grew crystals by vapour diffusion of diethyl ether into a concentrated solution of **1** in dimethylformamide and obtained samples suitable for X‐ray diffraction (Figure 1 a,b).

The compound itself showed an interesting electrochemical behaviour at a glassy carbon electrode. Two quasi‐reversible redox couples can be observed, and these have been related to the reduction and oxidation of the central Mn atom (Mn^III^⇄Mn^II^ and Mn^III^⇄Mn^IV^), a behaviour already found in amine‐ and TTF‐capped analogues.^42^ For the pyridyl‐capped POM **1**, the reduction process is very clearly electrochemically irreversible, with a peak‐to‐peak separation of ≈600 mV at low scan rates. The oxidation process is less irreversible, with a reduced peak‐to‐peak separation of ≈180 mV (see the Supporting Information for more details).

We then fabricated junctions using the scanning‐tunnelling‐microscopy break‐junction (STM‐BJ) method^43^ by driving a Au tip towards a Au substrate under constant DC bias until it crashes and forms a metallic contact with a conductance >5 *G*_0_ (*G*_0_=conductance quantum, 2 *e* ^2^/*h*≈77.48 μS). The tip is then withdrawn at constant speed, and the metallic contact is thinned down to a single atom (conductance=*G*_0_) and then broken. When the experiment is performed in a solution of a molecular wire having metal‐binding moieties at its termini, molecular junctions self‐assemble in the freshly formed nanogap. The process is repeated thousands of times while recording the current, and the conductance of the junctions is then calculated as *G*=*I*/*V*_BIAS_. The traces obtained in the process (as current vs. electrode position) are then analysed statistically as histograms and density maps to calculate the most probable conductance value. The experiments were performed under electrochemical control, using a bipotentiostat and a four‐electrode cell, with the Au substrate as the working electrode, a platinum counter electrode, and a platinum reference electrode (Figure 1 e). This setup was found to have an open‐circuit potential of −0.23 V vs. Fc/Fc^+^. The STM tip was coated with Apiezon wax^44^ to reduce Faradaic currents and constantly biased against the working electrode by the bipotentiostat to ensure the two Au electrodes act as source and drain. A tip–substrate bias of 200 mV was used throughout this study as the smallest bias voltage to maximise the signal‐to‐noise ratio. The measurements were performed with the four electrodes immersed in the ionic liquid BMIM–OTf (Figure 1 c) with a 1 mm concentration of compound **1**. The use of ionic liquids ensures a high degree of molecule–gate coupling^10^ and they are an ideal medium for STM experiments and measurements, with low Faradaic current attainable with insulated tips and a wide electrochemical window.^45^, ^46^, ^47^ More details about the STM‐BJ experiments, the electrochemistry of **1** in ionic liquids, and the equipment used in this study can be found in the Supporting Information.

**Figure 2:**
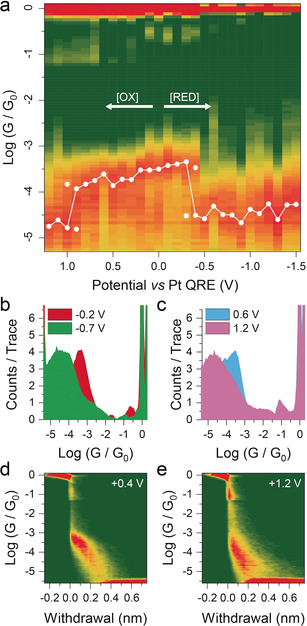
Single‐molecule electrochemical gating experiments on **1**. a) Heat map of conductance across the electrochemical window explored, with (overlaid in white) the Gaussian fitting of the conductance peaks. Double points are shown in the vicinity of the switching potential. b) Conductance histograms for compound **1** at an electrochemical potential of −0.2 V and −0.7 V. c) Conductance histogram of **1** at 0.6 V and 1.2 V. d) 2D conductance‐vs.‐electrode‐separation density map at 0.4 V. e) 2D conductance‐vs.‐electrode‐separation density map at 1.2 V. Heat map in (a) compiled with 10 bins per conductance decade. Conductance histograms in (b) and (c) compiled with 20 bins per conductance decade. 2D density maps in (d) and (e) compiled with 100 bins per conductance decade and 50 bins per nanometre. More than 3500 individual breaking traces have been collected at each potential. In all heatmaps and density plots: green=low counts and red=high counts. The full dataset is presented in the Supporting Information.

The main results are shown in Figure 2. We explored the bias window between 1.2 V and −1.5 V vs. Pt, which is attainable in the ionic liquid we used and where **1** exhibited three distinct charge states. As can be observed in Figure 2 a, when the electrochemical potential is moved from zero towards more negative values, there is a slight increase in conductance as the potential acts as an electrostatic gate and moves the Fermi level towards a transport resonance. However, when the potential reaches −0.3 V vs. Pt, the cluster accepts an electron and its charge state switches^42^, ^48^ to −4, with a strong effect on the conductance that drops by more than one order of magnitude, from 10^−3.4^ *G*_0_ to 10^−4.6^ *G*_0_. Example conductance histograms pertinent to the −3/−4 switch are given in Figure 2 b. Close to the equilibrium potential, both charge states are present, and they almost equally contribute to the conductance histogram (see all histograms in the Supporting Information and later in the manuscript for the analysis). As the potential gets more negative, the electrostatic‐gate effect is reinstated, and conductance gradually climbs to 10^−4.3^ *G*_0_. On the contrary, as the potential gets more positive than zero, the conductance gradually decreases due to the electrostatic‐gate effect until the charge state of the POM switches to −2 and its value falls to almost 10^−5^ *G*_0_. The effect of the −2/−3 switch on the conductance histograms can be seen in Figure 2 c. Statistical analysis of the conductance‐electrode separation traces as 2D density plots (see, for example, Figure 2 d,e and the Supporting Information for the full dataset) shows that junction‐rupture length remains constant at approximately 0.4–0.5 nm, as **1** is oxidised or reduced. Significant structural or configurational rearrangements as the oxidation state of **1** is switched can therefore be discounted. Accounting for the electrode snapback (0.65 nm at room temperature),^7^ we can calculate a junction length at rupture of 1.05–1.15 nm, only slightly shorter than the molecular N−N distance obtained from single‐crystal X‐ray diffraction (SCXRD; 1.4 nm).

Two main things can be inferred from the above results. First, the observed electrostatic gate effect, where conductance increases as the potential gets more negative, strongly suggests that charge transport happens through a non‐resonant one‐step tunnelling mechanism assisted by the molecular LUMO (pyridyls are known^36^ to promote LUMO‐based conductance). Second, the oxidation and reduction of the central Mn atom have a profound effect on the electronic structure of the POM, as the conductance is electrochemically gated by approximately an order of magnitude in both cases. The confirmation of a tunnelling mechanism, and the clear absence of a bell‐shaped conductance enhancement at the equilibrium potential, discount a possible interpretation based on a two‐step Kuznetsov–Ulstrup process (a hopping‐type model associated with a conductance increase near the redox potential).^49^, ^50^ A Nernstian model based on charge tunnelling through different redox states of a molecule (in thermodynamic equilibrium depending on the applied electrochemical potential) has been developed to aid the interpretation of data obtained from junctions based on anthraquinone‐substituted DNA^51^ or electroactive ferrocenes.^52^ In ferrocenes, which lie closer in size to our present system, the mechanism of charge transport was unambiguously confirmed to be phase‐coherent tunnelling by analysis of the conductance decay with length, and the average conductance of the junction under electrochemical control is given by the equation:

(1) $$\bar{G}=G_{1}+\frac{1}{1+exp\left[e/k_{B}T\left(E-E_{0}\right)\right]}\left(G_{1}-G_{2}\right)$$

**Figure 3:**
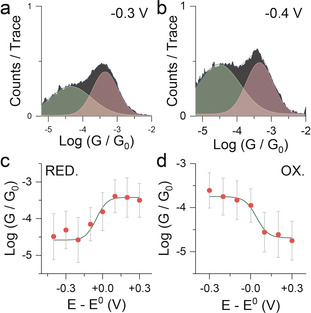
Gaussian fitting and Nernstian model. Examples of Gaussian fitting of conductance histograms of **1** at an electrochemical potential of a) −0.3 V and b) −0.4 V, with the background‐tunnelling signal subtracted, used for the calculations of *G_n_* and *S_n_*. Further details on the background‐subtraction process are available in the Supporting Information. c) Average conductance (orange dots) vs. equilibrium potential for the reduction process (*E*_0_=−0.3 V vs. Pt). d) Average conductance (orange dots) vs. equilibrium potential for the oxidation process (*E*_0_=0.9 V vs. Pt). The grey line in (c) and (d) is the Nernstian model obtained from Equation (1). Histograms in (a) and (b) compiled with 100 bins per decade.

Here, *G*_1_ and *G*_2_ are the conductance of the molecular junction in the higher and lower oxidation states, respectively, *e* is the electron charge, *k*_B_ is the Boltzmann constant, *T* is the temperature, *E* is the electrochemical potential, and *E*_0_ is the equilibrium potential. We calculated $\bar{G}$ as the weighted arithmetic mean of the Gaussian fitting of the conductance histograms:

(2) $$\bar{G}=\;\frac{G_{1}A_{1}+G_{2}A_{2}}{A_{1}+A_{2}}$$

using the peak centre as the value for *G* and the area under the Gaussian curve as *A*, after removal of the background tunnelling signal obtained from a control experiment performed in the pure, anhydrous solvent. The weighted average of the experimental points and the Nernstian model are in good agreement, as can be seen in in Figure 3. It is worth stressing that the Nernstian model is not a fitting to the experimental conductance values, as no variable was parameterised. The agreement between Equation (1) and the empirical data thereby confirms non‐resonant tunnelling as the dominant charge‐transport mechanism, with the molecule either reduced or oxidised in the junction, depending on the potential.

**Figure 4:**
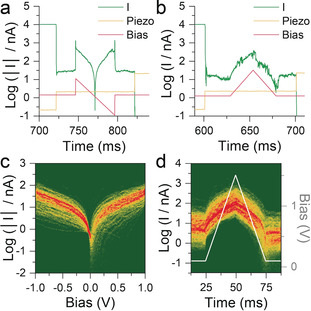
Two‐terminal bias‐modulation experiments. Example a) raw *I*/*V* data (−1 to 1 V sweep) and b) raw high bias ramp curve (0.1 to 1.5 to 0.1 V sweep), with piezo and bias signal superimposed. c) 2D density map of the *I*/*V* characteristics for compound **1**, compiled from 1049 traces. d) 2D density map of current vs. time during the high‐voltage ramp (superimposed in light grey), compiled from 922 traces. 2D maps in (c) and (d) compiled with 100 bins per current decade, 200 bins V^−1^, and 2000 bins s^−1^.

To further test the robustness of the junctions fabricated with the polyoxometalate **1**, we performed voltage ramps on stabilised junctions in a two‐terminal device configuration. In this technique, a staircase ramp is applied to the piezoelectric tube controlling the tip position, so that a specific position is held for 100 ms. The height of the staircase (1 nm) was chosen to ensure the formation of a gap of a size commensurate with the molecular length. During the hold portion of the ramp, the bias voltage is kept at a fixed value (100 mV) for 25 ms, then ramped to obtain *I*/*V* characteristics or a high‐voltage test, and then kept again at the fixed value for the final 25 ms (Figure 4 a). Data is then sliced and processed using the automated algorithms described in the Supporting Information to produce the 2D density maps presented in Figure 4 c,d. The *I*/*V* characteristics show a good ohmic behaviour up to relatively high biases (≈0.7 V, see the Supporting Information for linear‐scale plots) with no evident asymmetry or rectification arising from the presence of a molecular orbital with an energy sufficiently close^53^, ^54^ to the Fermi levels of the gold electrodes under the two‐terminal conditions employed. This further confirms off‐resonant tunnelling as the charge‐transport mechanism, and this would also account for the observed relatively low conductance. As a stress test, we drove the junctions to biases higher than the one used to obtain the *I*/*V* behaviour, and we found excellent robustness under positive substrate bias, with the fabricated junctions surviving voltages up to 1.5 V.

## Conclusion

In conclusion, we have demonstrated the use of a functionalised Anderson–Evans polyoxometalate in a three‐state electrochemical transistor single‐molecule device. The MnMo_6_O_24_ oxide‐cluster core is capped with two 4‐pyridyl moieties that act as contacts to the electrodes, allowing the fabrication of single‐POM junctions. The junctions showed a clear OFF‐ON‐OFF behaviour under electrochemical control, with a difference in conductance of more than one order of magnitude between two adjacent charge states. The transistor‐like behaviour arises from the POM being in three distinct charge states (−2, −3, and −4) depending on the applied electrochemical potential, and a clear electrostatic gating is further visible in the conductance‐vs.‐potential plot. The results can be modelled within the framework of non‐resonant tunnelling, with the charge states having a different tunnelling probability depending on the charge state of the molecular wire. The fabricated junctions are robust even under high bias, further showing the promise that POMs have in the field of molecular electronics, thanks to their multiple charge/oxidation states and high stability. Our results pave the way to a further use of such compounds in cluster electronics.^55^

## Conflict of interest

The authors declare no conflict of interest.

## Supporting information

As a service to our authors and readers, this journal provides supporting information supplied by the authors. Such materials are peer reviewed and may be re‐organized for online delivery, but are not copy‐edited or typeset. Technical support issues arising from supporting information (other than missing files) should be addressed to the authors.

Supplementary
