# Supplementary material for: A Chemically Soldered Polyoxometalate Single‐Molecule Transistor
Source: Angew Chem Int Ed Engl. 2020 May 12;59(29):12029–34. doi: 10.1002/anie.202002174 (PMC7383859; doi:10.1002/anie.202002174)
Supplement: Supplementary file 1 — Supplementary [file ANIE-59-12029-s001.pdf]

Angewandte Chemie
Eine Zeitschrift der Gesellschaft Deutscher Chemiker

Supporting Information

A Chemically Soldered Polyoxometalate Single-Molecule Transistor**

*Chuanli Wu, Xiaohang Qiao, Craig M. Robertson, Simon J. Higgins, Chenxin Cai, Richard J. Nichols, and Andrea Vezzoli**

anie_202002174_sm_miscellaneous_information.pdf

Author Contributions

C.W. Formal analysis: Supporting; Investigation: Lead; Methodology: Supporting; Writing—Review & Editing: Supporting
X.Q. Formal analysis: Supporting; Investigation: Supporting; Methodology: Supporting
C.R. XRD crystal structure collection and analysis: Lead
S.H. Formal analysis: Supporting; Validation: Supporting; Writing—Review & Editing: Supporting
C.C. Funding acquisition: Supporting; Supervision: Supporting; Validation: Supporting
R.N. Formal analysis: Supporting; Funding acquisition: Lead; Supervision: Equal; Validation: Supporting; Writing—Review & Editing: Supporting
A.V. Conceptualization: Lead; Data curation: Supporting; Formal analysis: Lead; Funding acquisition: Supporting; Investigation: Supporting; Methodology: Supporting; Software: Lead; Supervision: Equal; Writing—Original Draft: Lead; Writing—Review & Editing: Lead.

Contents

1. Methods..2

2. Materials and synthetic procedures 3

2.1 Synthesis of **1** 3

3. Details on *STM-BJ* Measurements. 4

3.1. *STM-BJ* under electrochemical control 4

3.2 *STM-BJ* under bias modulation 4

3.3 Bias modulation data analysis 5

4. Additional *STM-BJ* Data 7

4.1 Raw *STM-BJ* conductance histograms...7

4.2 *STM-BJ* conductance – electrode withdrawal density maps 8

4.3 Details on background subtraction for Nernstian plots...9

4.4 Histograms after background subtraction 10

4.5 Linear scale I/V characteristics..11

4.6 Control experiment on 4-tris(hydroxymethyl)pyridine..11

5. Details on the electrochemistry of **1** 12

6. Crystal data for **1** 15

7. References 31

1. Methods

Compound **1** was prepared following a published procedure.[1] Junctions were fabricated using the *STM-BJ* technique,[2] using a modified Keysight 5500 electrochemical STM. Measurements were performed in 1 mM solutions of **1** in the ionic liquid 1-butyl-3-methylimidazolium trifluoromethanesulfonate (BMIM-OTf, IoLiTec GmbH). The ionic liquid was dehydrated by heating for >16 hours at 120 °C *in vacuo* (~ 8 mbar) in the presence of 4Å molecular sieves. Measurements were performed using an Au tip (99.99+%, Goodfellow Cambridge Ltd) insulated with wax (Apiezon Wax W40, M&I Materials Ltd). Substrates were prepared by thermal evaporation of Au (99.99+%, Goodfellow Cambridge Ltd) on freshly cleaved muscovite mica (Agar Scientific Ltd). Conductance histograms were compiled with no data selection using >3500 consecutively measured traces. Cyclic voltammetry was performed with a Metrohm Autolab PGSTAT 128N potentiostat, in a standard three electrode configuration.

Further details on the synthesis of **1** (including spectroscopic and crystallographic data), the instrumentation used, and the data collection and analysis processes can be found in this document.

2. Materials and synthetic procedures

All reagents were purchased from Sigma-Aldrich Chemical Company (now MilliporeSigma) except sodium molybdate dihydrate, which was purchased from Alfa Aesar. All solvents were purchased from ThermoFisher scientific except the ionic liquid BMIM-OTf, which was purchased from IoLiTec GmbH. Starting materials and solvents were used as received, except BMIM-OTf that was dried *in vacuo* over molecular sieves at 120 °C overnight.

^{1}H and ^{13}C spectra were acquired on a Bruker Avance 400 Ultrashield spectrometer and referenced to internal TMS and the residual solvent peak. Mass spectra were recorded using an Agilent Q-TOF 7200.

2.1 Synthesis of **1**

The synthesis of **1** as tris(tetrabutylammonium) salt was performed following a literature method.[1] In brief, we prepared tris(hydroxymethyl)methyl-4-pyridine by refluxing γ-picoline in 37% formaldehyde solution overnight, and $[NBu_4]_4[\alpha\text{-}Mo_8O_{26}]$ by adding tetrabutylammonium bromide to an acidified aqueous sodium molybdate solution.[3] tris(hydroxymethyl)methyl-4-pyridine and $[NBu_4]_4[\alpha\text{-}Mo_8O_{26}]$ were then refluxed overnight in acetonitrile with manganese(III)acetate dihydrate to give **1** in 71 % yield. 1H NMR (400 MHz, DMSO-d6): 63.67 (s, 12H), 8.16 (s, 4H), 7.02 (s, 4H), 3.16 (s, 24H), 1.57 (s, 24H), 1.32 (s, 24H), 0.93 (s, 36H). *m/z* (HRMS, CH_3OH, ES-): $[\mathbf{1}(NBu_4)_2]^-$ 1763.982 (*calc.* 1763.973); $[\mathbf{1}(NBu_4)+H^+]^-$ 1521.704 (*calc.* 1521.707). Further adducts with methanol are visible in the mass spectrum at lower intensities. Spectroscopic data is consistent with the values reported in the literature. Crystal data presented in section 5 of the SI.

3. Details on *STM-BJ* Measurements.

3.1. *STM-BJ* under electrochemical control

We used a commercial STM (Keysight Technologies 5500 SPM), equipped with a 4-channel custom preamp based on the design by Meszaros *et al.*[4] and a National Instruments NI9215 USB data acquisition board (16-bit, 10 KSa/s). A bipotentiostat (Keysight Technologies) is used to control a 4-electrode cell, consisting of the STM tip (cut from a spool of annealed 99.99+% Au wire, Goodfellow Cambridge Ltd and insulated with Apiezon wax) and the Au substrate (Au on mica) that act as working electrodes, a coiled platinum wire as counter electrode and a platinum wire as pseudo-reference electrode. A constant bias is maintained by the bipotentiostat between the Au tip and the Au substrate, while an electrochemical potential is kept between the Au substrate and the counter electrode, relative to the reference. All experiments have been performed at a bias of 200 mV (sample positive) in a 1 mM solution of compound **1** in BMIM-OTf.

As described in the main paper, the Au tip is driven towards the substrate to create a nanocontact having conductance $\gg G_0$, and then withdrawn at a constant speed of $20\ nm/s$. Current is monitored during the process and the conductance then calculated as $G = I/V$ as a function of G_0. Each cycle results in a current-distance trace, and thousands of these are acquired at each electrochemical potential and compiled in the plots (conductance histograms, heatmaps and density plots) shown in the main paper and here in the SI with no data selection.

As with all electrochemical break-junction measurements we monitor the “diffusion current”, also known as “tip leakage current”. The leakage current for the coated STM tips is monitored with the tip away from the surface before we do the BJ measurements. We only proceed if the leakage current is much smaller than the molecular junction current, since single molecule measurements cannot be made when there is significant leakage. In addition, before performing the measurements in the ionic liquid BMIM-OTf, we performed measurements in other media, such as a propylene carbonate solution and in air after deposition of a sub-monolayer on a Au substrate, and found very similar values of conductance.

3.2 *STM-BJ* under bias modulation

We used a commercial STM (Keysight Technologies 5500 SPM) equipped with a Femto DLPCA-200 preamplifier and a PXI-4464 data acquisition board (24-bit, operating at 20 kHz). An arbitrary waveform generator (Keysight Technologies 33522B) was used to apply voltage ramps to the piezo and the sample bias, through the STM breakout box (Keysight Technologies N9477A). Experiments were performed in a two-terminal configuration, with an STM tip (cut from a spool of annealed 99.99+% Au wire, Goodfellow Cambridge Ltd. and insulated with Apiezon wax) and the Au substrate (Au on mica) in a 1 mM solution of compound **1** in BMIM-OTf.

As described in the main paper, a staircase ramp is applied to the piezo, with abrupt stretches of 1 nm and 100 ms “hold” portions. Between stretches, the voltage is held fixed at 0.1 V for 25 ms, then modulated according to the ramps shown in Figure 4 of the main paper for 50 ms, and then held fixed at 0.1 V for the

last 25 ms. For the I/V experiments, bias was swept between 1 and -1 V at a rate of 40 V/s, while for the high bias test the bias was swept as 0.1 V → 1.5 V → 0.1 V at a rate of 56 V/s.

3.3 Bias modulation data analysis

An automated algorithm was used to analyse the data obtained under bias modulation. First, the algorithm slices the trace between stretches by analysing the signal imposed to the piezo and cutting where its second derivative is > 0.1 (Figure S1).

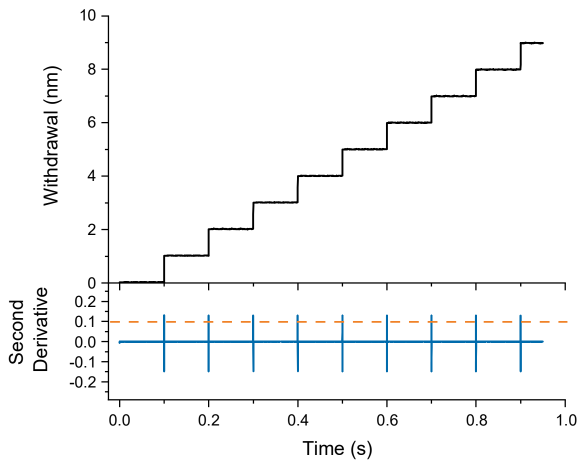

Figure S1: Example of piezo signal of a bias modulation experiment (in nm) with its second derivative, used to slice the trace in between abrupt stretches. The threshold of 0.1 is shown as red dashed line.

The transimpedance amplifier signal is then converted to current by applying the conversion factor used in the experiment (either 10^6 or 10^7 V/A), and the resulting slices are fed into a sorting algorithm, that calculates the average of the conductance in a portion of the first and last 25 ms of the slice and checks that both are within the experimentally determined confidence interval. This filters out traces where no molecule is bridging the gap, those in which the tip is in contact with the substrate and those where the molecular bridge did not survive the bias modulation process. The resulting slices, relative to the bias modulation of a stable molecular junction (Figure S2), are compiled with no further processing in the 2d density maps shown in the main paper.

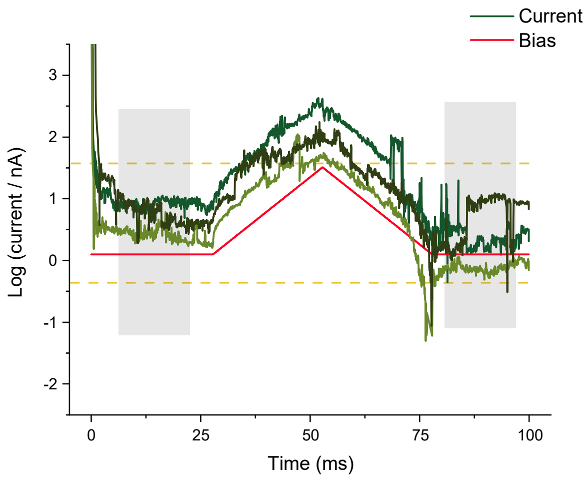

Figure S2: Example of three slices obtained during the high voltage experiment. The average value of the current in the grey boxes must be within the two limits shown as yellow dotted line for a trace to be saved by our automated algorithm.

In total, of the 3423 raw I/V curves, the algorithm saved 1049 slices (30%), and of the 3421 high bias traces, the algorithm saved 922 slices (27%).

4. Additional *STM-BJ* Data

4.1 Raw *STM-BJ* conductance histograms

All the histograms obtained under electrochemical control are shown stacked below (Figure S3).

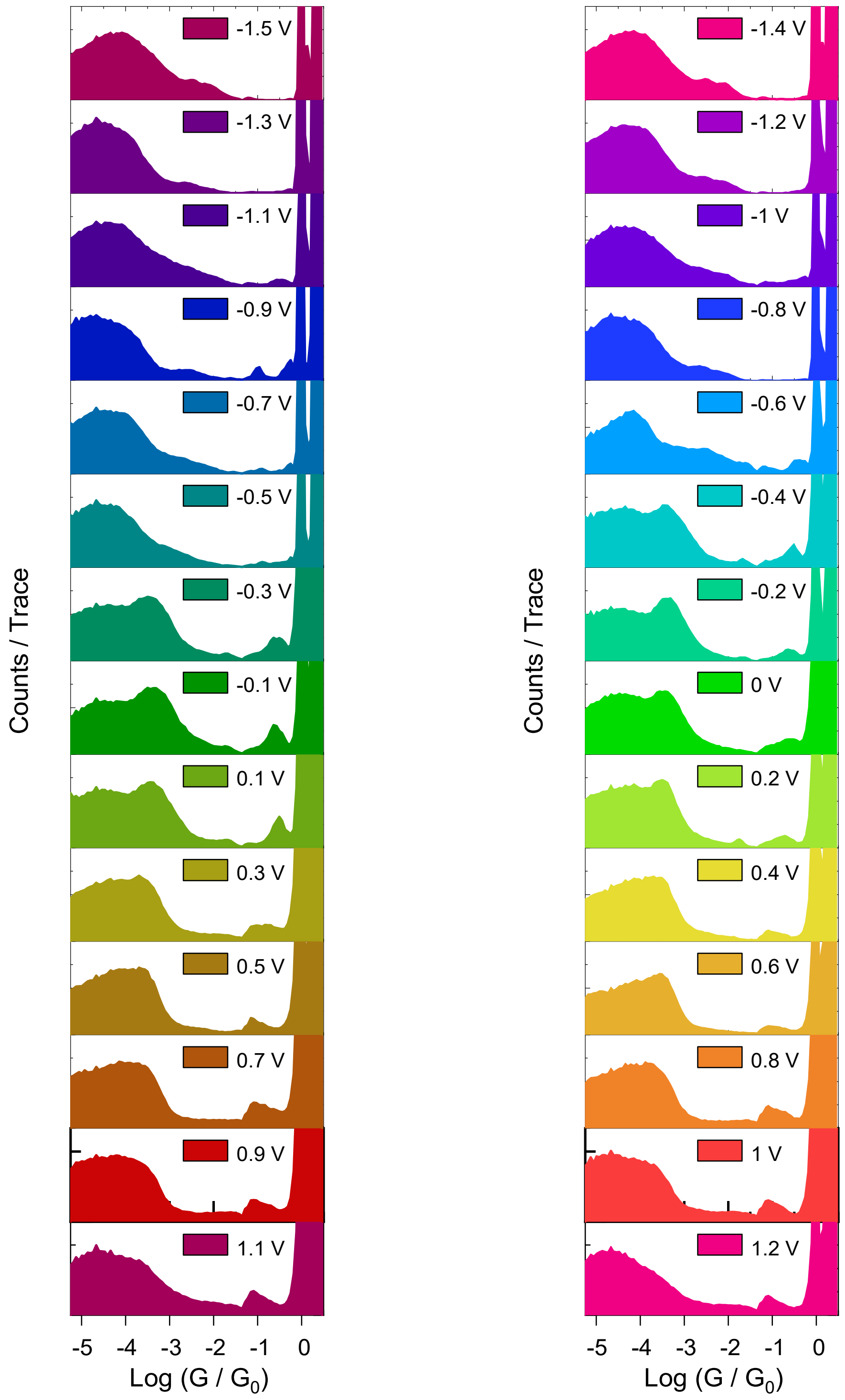

Figure S3: Raw conductance histograms for compound **1** at all the electrochemical potentials shown in Figure 2 of the main paper. Histograms compiled with 20 bins per conductance decade, and normalised as counts/trace to the number of traces used. The electrochemical potential is included as legend. All histograms contain > 3500 individual traces. All data obtained at 200 mV tip-sample bias.

4.2 *STM-BJ* conductance – electrode withdrawal density maps

All the 2D density maps obtained under electrochemical control are shown below (Figure S4).

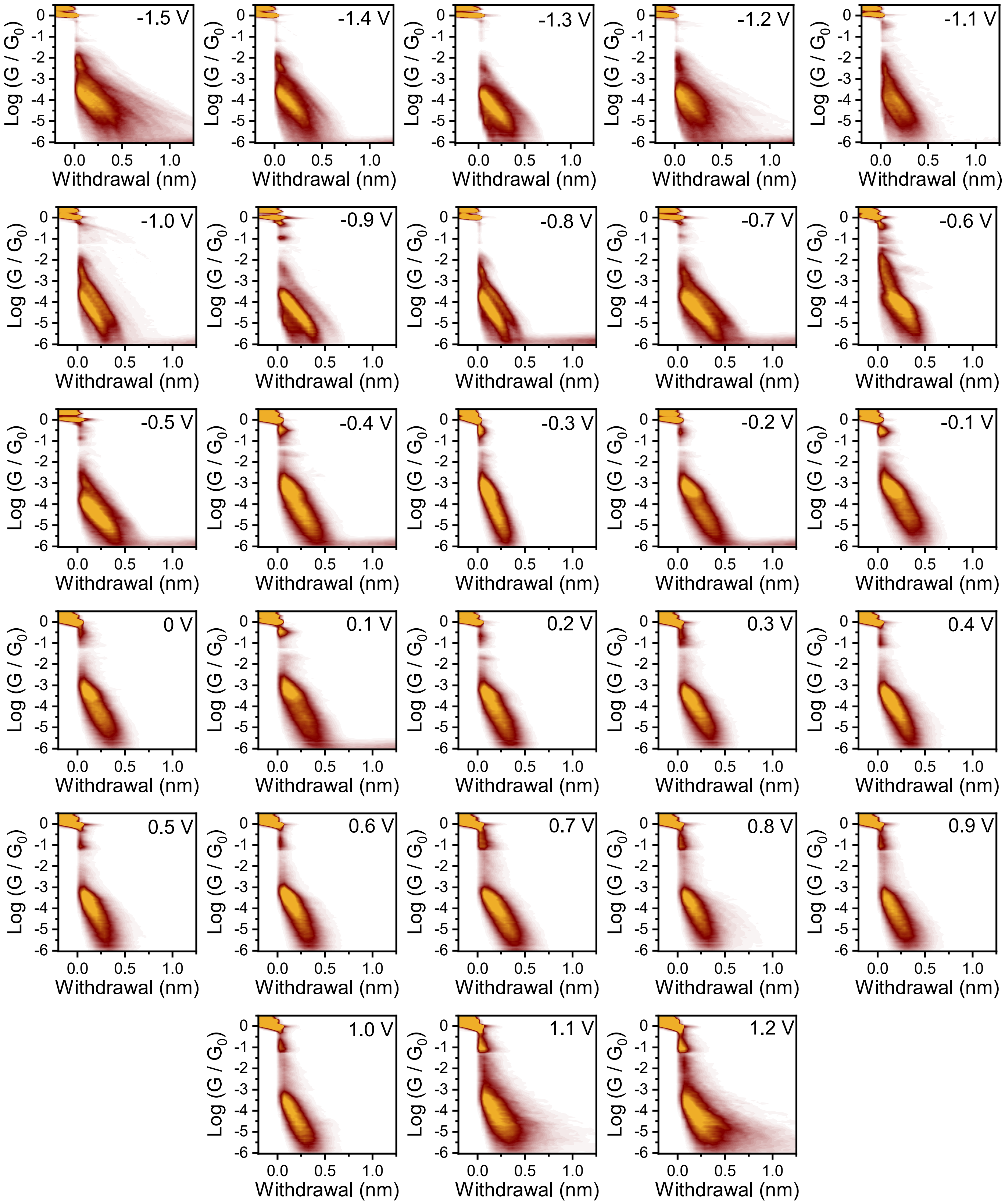

Figure S4: Two-dimensional conductance vs electrode withdrawal density maps for **1**. The electrochemical potential (vs Pt quasi-reference) used in the experiment is written in the inset. All maps compiled with 15 bins per conductance decade and 30 bins per nanometre. Colour scale normalised to the number of scans used to compile the map. White = no counts, increasing to orange = max (0.6 counts / trace). All density maps contain > 3500 individual traces obtained at 200 mV tip-sample bias.

4.3 Details on background subtraction for Nernstian plots

The low conductance peak appearing upon reduction or oxidation of the polyoxometalate is at $10^{-5} – 10^{-4}$ G_0, where there is a significant tunnelling contribution arising from the *STM-BJ* traces where no molecular junction is in place. To avoid overestimating its contribution, we performed a background subtraction, using data from a measurement in pure BMIM-OTf. Examples of the resulting histograms, in the range $10^{-2} – 10^{-5}$ G_0 are shown in the main paper, and the process is shown in Figure S5.

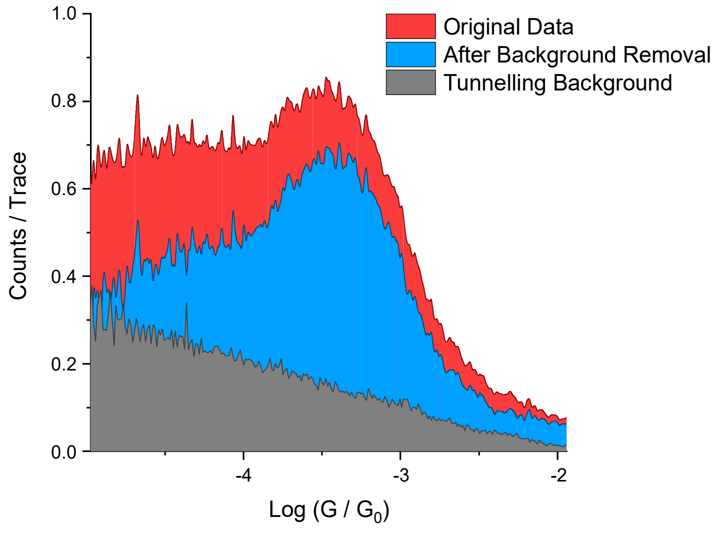

Figure S5: Example of background removal to obtain the plots shown in Figure 3 of the main paper. The resulting blue histogram was then used to obtain the Gaussian fittings for the Nerstian plot. 100 bins per conductance decade.

We used the same binning for the background subtraction (100 bins per decade) and normalised each histogram to the number of traces used to compile it. All histograms after background subtraction are shown below.

4.4 Histograms after background subtraction

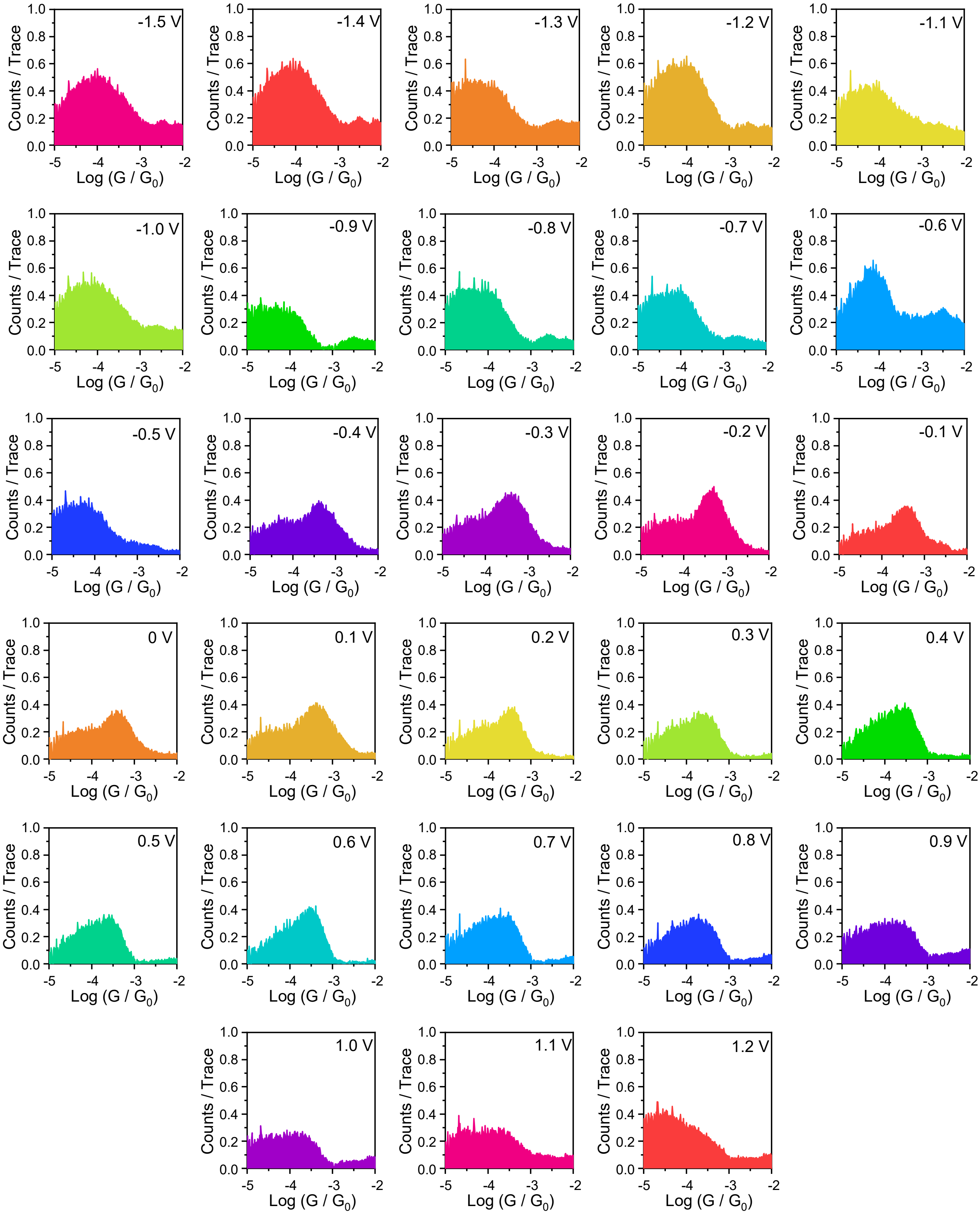

Figure S6: Conductance histograms of **1** under electrochemical control after background subtraction. The electrochemical potential (vs Pt quasi-reference) used in the experiment is written in the inset. All plots compiled with 100 bins per conductance decade and normalised as counts/trace to the number of traces used (>3500 each). Data obtained at 200 mV tip-substrate bias.

4.5 Linear scale I/V characteristics

In addition to the logarithmic I/V characteristics density map shown in the main paper, we present here the linear scale version (Figure S7), along with the calculated average.

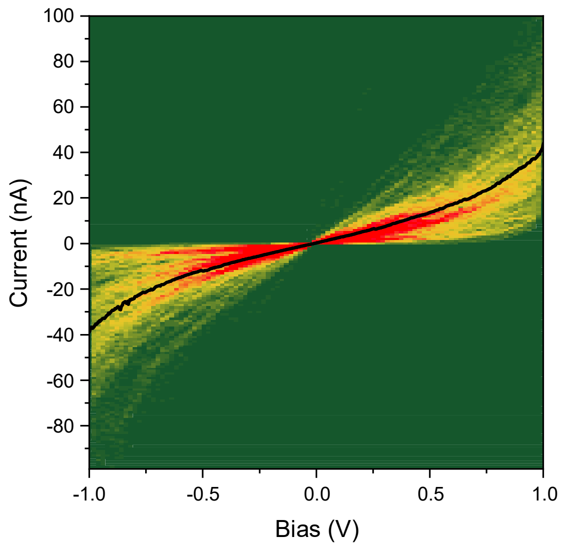

Figure S7: Linear scale I/V density map. The calculated average curve is superimposed as black line. 2D map compiled with 1 bin per nA and 50 bins per volt. 1049 traces have been used in the map and averaged to obtain the black line.

4.6 Control experiment on 4-tris(hydroxymethyl)pyridine

In order to establish that the ligand itself cannot form junctions and that the data presented so far does not arise from a breakdown of **1** into its components, we performed *STM-BJ* measurements on 4-tris(hydroxymethyl)pyridine (Figure S8). No evidence of junction formation was found, and only a raised low-conductance noise was observed (~0.4 counts/trace vs ~0.3 counts/trace observed in pure solvent, Figure S5), possibly due to 4-tris(hydroxymethyl)pyridine adsorbed on only one of the electrodes through its pyridyl end.

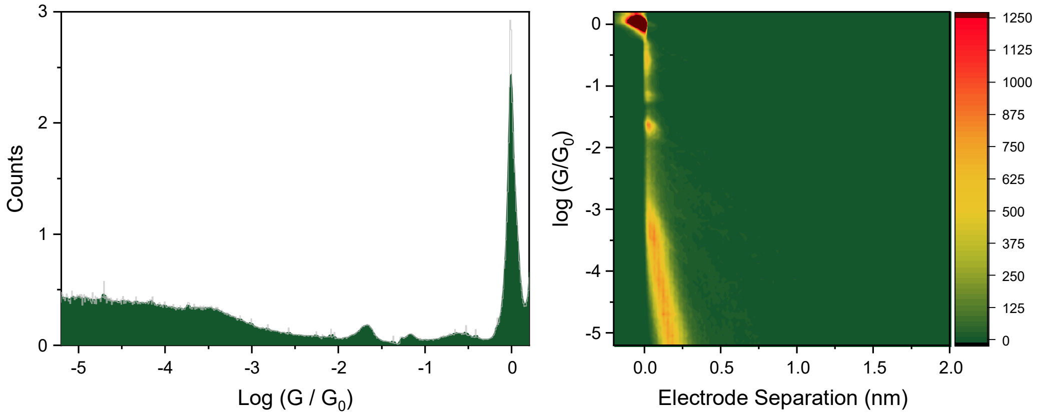

Figure S8: *STM-BJ* measurements on 4-tris(hydroxymethyl)pyridine in BMIM-OTf (1 mM). No discernible peaks can be observed in the histogram (right) in the region where **1** shows conductance features, and no signal extends by more than 0.2 nm in the 2D density map (left). Data compiled from 4642 scans, 100 bins per conductance decade and 100 bins per nanometre.

5. Details on the electrochemistry of **1**

A Metrohm Autolab PGSTAT 128N potentiostat in a standard three electrode configuration was used for all electrochemical measurements. We used a 3 mm diameter glassy carbon (GC) electrode (IJ Cambria Scientific Ltd) as working electrode and freshly annealed coiled platinum wires as counter electrode and reference electrode. The GC electrode was polished with diamond slurries of decreasing size (Buehler) on a silk-type cloth pad (Kemet International) to a mirror finish, then washed with Milli-Q water, briefly sonicated in Milli-Q water, rinsed in acetone and dried under a stream of nitrogen.

The electrochemical characterisation (Figure S9) was performed in dimethylformamide to ensure good solubility, using tetrabutylammonium tetrafluoroborate as support electrolyte (0.1 M).

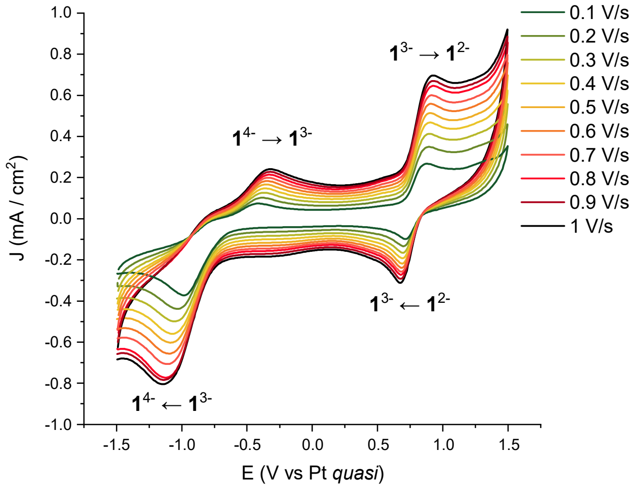

Figure S9: Cyclic voltammetry (glassy carbon electrode) of **1** in DMF with 0.1M NBu_4BF_4 as supporting electrolyte, at different scan rates.

Both redox waves exhibit cathodic and anodic peaks in the reverse scan, indicating that **1** can be reduced and re-oxidised. Good linearity was also found in the cathodic peak height *vs* (scan rate)$^{1/2}$ plot (Randles-Sevcik, Figure S10)

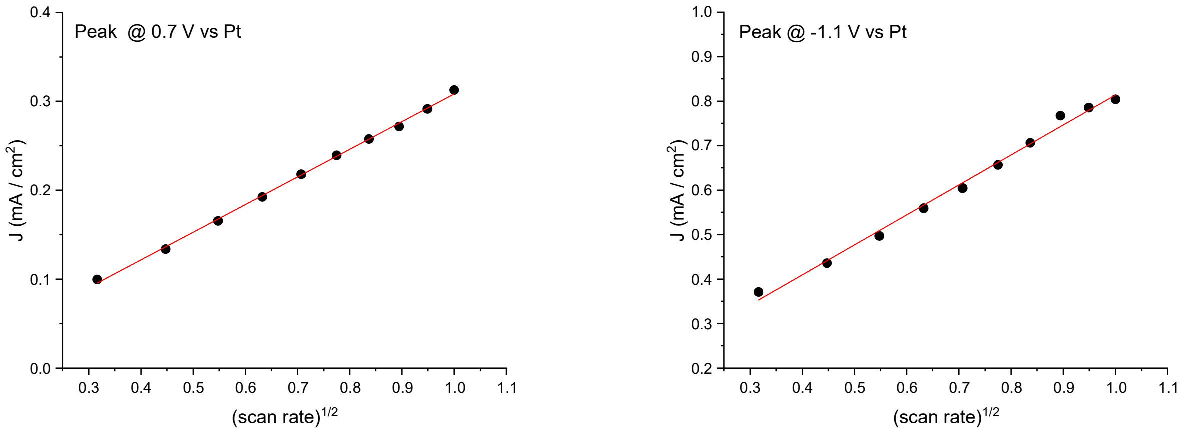

Figure S10: Peak height *vs* square root of the scan rate (Randles-Sevcik) plots for the cathodic peaks at approx. 0.7 V and -1.1 V. The red line is the linear fit of data.

The plot shown in Figure 1c of the main paper is referenced to Fc/Fc$^+$, using ferrocene as internal standard that was added at the end of the data acquisition process. The CV used for calibration are shown below (Figure S11).

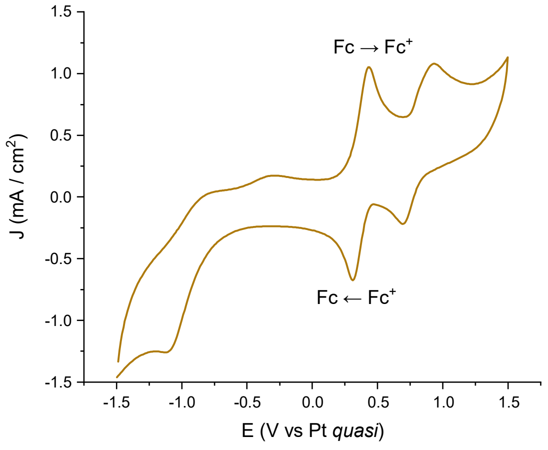

Figure S11: Cyclic voltammetry (glassy carbon electrode) of **1** in DMF with 0.1 M NBu_4BF_4 as supporting electrolyte, and ferrocene added as internal calibration, at a scan rate of 0.5 V/s

Repeating the same CVs in BMIM-OTf (Figure S12) gave less clearly defined peaks, mostly due to the low diffusion coefficient in the ionic liquid and a limited solubility of **1**. Indeed, achieving concentrations higher than 1-2 mM proved impossible, even after long sonication. Nevertheless, the redox pairs observed in DMF are still visible, and the reversibility of the $Mn^{III} \rightleftarrows Mn^{II}$ transition is improved.

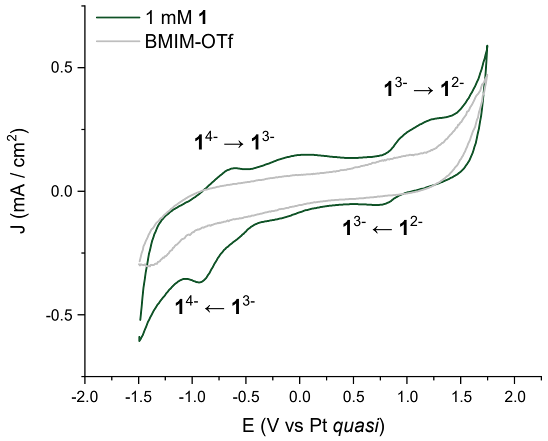

Figure S12: Cyclic voltammetry (glassy carbon electrode) of **1** in BMIM-OTf using a glassy carbon electrode, and background solvent signal at a scan rate of 0.5 V/s.

To have a comparison with the *in-situ* electrochemistry performed during an *STM-BJ* experiment, we also performed *ex-situ* cyclic voltammetry on a gold working electrode (Figure S13). Results are similar to the GC electrode. We used a 1 mm diameter Au electrode (IJ Cambria Scientific Ltd) as working electrode, a platinum mesh as counterelectrode and freshly annealed coiled platinum wires reference electrode. The Au electrode was polished with diamond slurries of decreasing size (Buehler) on a silk-type cloth pad (Kemet International)

to a mirror finish, then washed with Milli-Q water, briefly sonicated in Milli-Q water, rinsed in acetone and dried under a stream of nitrogen.

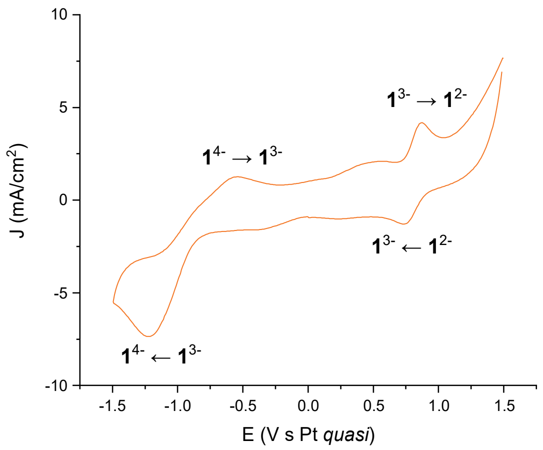

Figure S13: Cyclic voltammetry (Au disk electrode) of **1** in DMF with 0.1 M NBu_4BF_4 as supporting electrolyte at a scan rate of 1 V/s.

6. Crystal data for **1**

A single crystal of **1** was selected and mounted in parabar oil on a MiteGen tip and placed on a 'Bruker D8 Venture' diffractometer. The crystal was kept at 150 K during data collection. Using Olex2[5], the structure was solved with the ShelXT[6] structure solution program using Intrinsic Phasing and refined with the ShelXL[7] refinement package using Least Squares minimisation.

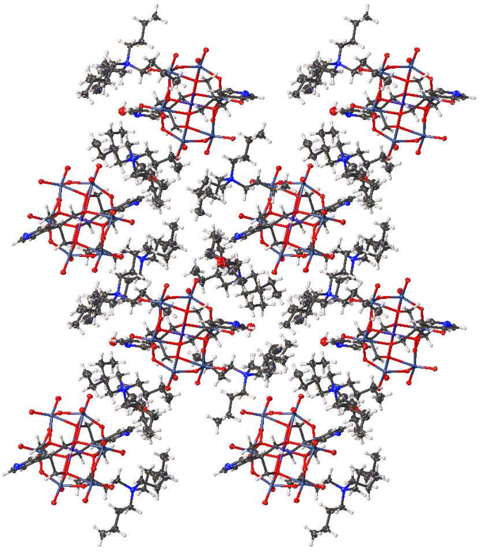

Figure S14: Portion of the SCXRD solid-state structure of **1** with counterions (NBu_4^+) and solvents along the <100> crystallographic plane. Legend: C = grey; H = white; Mn = purple; Mo = light blue; N = dark blue; O = red.

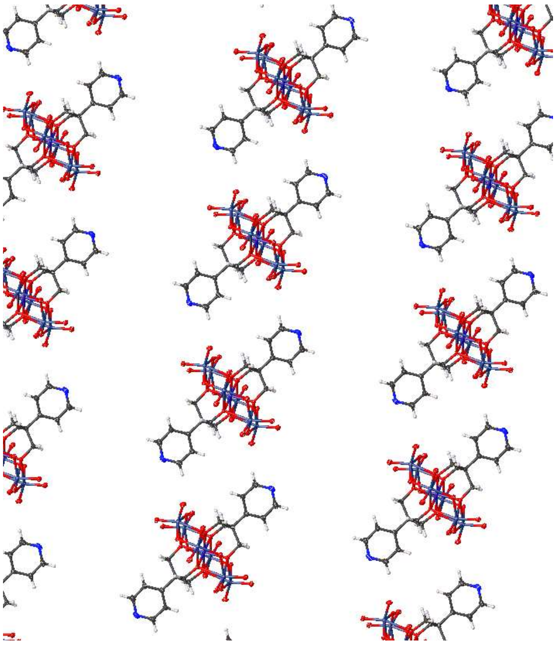

Figure S15: Portion of the SCXRD solid-state structure of **1** along the <001> crystallographic plane. Counterions and solvent molecules not shown for clarity. Legend: C = grey; H = white; Mn = purple; Mo = light blue; N = dark blue; O = red.

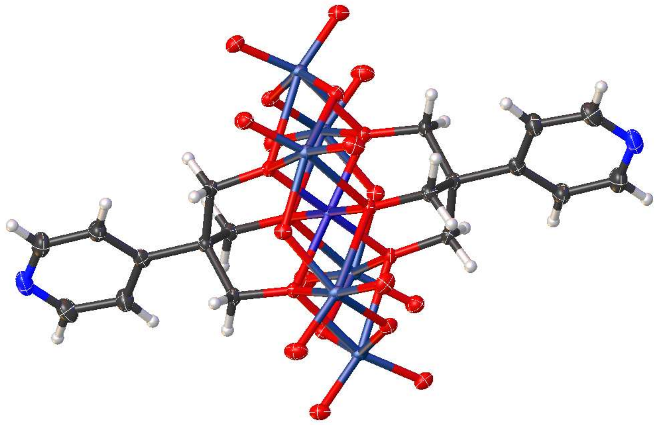

Figure S16: Magnification of the SCXRD structure of **1** along the <111> crystallographic plane. Counterions and solvent molecules not shown for clarity. Legend: C = grey; H = white; Mn = purple; Mo = light blue; N = dark blue; O = red.

Empirical formula	$C_{70}H_{137}MnMo_6N_5O_{25.5}$
Formula weight	2087.42
Temperature/K	150
Crystal system	triclinic
Space group	P-1
a/Å	12.9111(12)
b/Å	18.2011(18)
c/Å	19.6030(16)
α/°	83.907(3)
β/°	76.265(2)
γ/°	73.172(2)
Volume/Å^3	4279.8(7)
Z	2
ρ_{calc}g/cm^3	1.620
μ/mm^{-1}	1.067
F(000)	2146.0
Crystal size/mm^3	0.16 × 0.15 × 0.02
Radiation	MoKα (λ = 0.71073)
2Θ range for data collection/°	3.106 to 52.874
Index ranges	-16 ≤ h ≤ 16, -22 ≤ k ≤ 22, -24 ≤ l ≤ 24

Reflections collected	118631
Independent reflections	17584 [R_{int} = 0.0620, R_{sigma} = 0.0392]
Data/restraints/parameters	17584/105/1036
Goodness-of-fit on F^2	1.041
Final R indexes [I>=2σ (I)]	R_1 = 0.0350, wR_2 = 0.0761
Final R indexes [all data]	R_1 = 0.0498, wR_2 = 0.0831
Largest diff. peak/hole / e Å^{-3}	1.31/-0.74

Table S1: Fractional Atomic Coordinates (×10^4) and Equivalent Isotropic Displacement Parameters (Å^2×10^3) for **1**. U_{eq} is defined as 1/3 of of the trace of the orthogonalised U_{IJ} tensor.

Atom	*x*	*y*	*z*	U(eq)
Mo1B	-1789.9(2)	5460.2(2)	1554.2(2)	15.82(7)
Mo3B	2242.7(2)	3504.3(2)	-80.3(2)	15.85(7)
Mo1A	6056.2(2)	11405.5(2)	5242.4(2)	14.21(7)
Mo2A	5899.5(2)	11076.5(2)	3611.1(2)	14.30(7)
Mo2B	480.8(2)	3986.7(2)	1478.3(2)	15.64(7)
Mo3A	4849.8(2)	9675.2(2)	3371.0(2)	15.36(7)
Mn1B	0	5000	0	12.39(14)
Mn1A	5000	10000	5000	10.81(14)
O2B	-285.7(18)	6065.8(13)	-282.7(11)	15.0(5)
O1B	76.7(18)	5218.9(13)	1015.7(11)	15.0(5)
O4B	-1627(2)	5750.7(14)	2311.6(12)	21.7(5)
O6A	6733.9(18)	11050.1(13)	4308.5(12)	15.6(5)
O7B	726.0(19)	4262.6(14)	2212.9(12)	21.5(5)
O2A	5296.6(18)	8917.5(12)	5213.2(11)	13.7(5)
O6B	-1101.2(18)	4370.0(13)	1593.4(12)	17.5(5)
O4A	7225(2)	11306.2(14)	5543.0(12)	21.8(5)
O3B	1577.2(18)	4818.4(13)	-338.9(11)	14.7(5)
O11B	1851.5(18)	3611.4(13)	-979.3(12)	17.7(5)
O10B	3602.6(19)	3497.4(14)	-303.9(12)	22.8(6)
O11A	3646(2)	9769.3(14)	3109.2(13)	22.7(6)
O12A	4992.0(19)	8711.0(13)	3903.9(11)	16.2(5)
O12B	2230(2)	2578.3(14)	134.6(13)	23.1(6)
O9A	4717.1(19)	10751.3(13)	3407.1(11)	16.7(5)
O1A	6151.1(18)	10093.8(12)	5438.0(11)	14.4(5)
O5B	-3133.2(19)	5424.8(14)	1712.8(13)	23.0(6)
O8A	6940(2)	10829.0(14)	2894.1(12)	21.2(5)
O10A	5881(2)	9413.5(14)	2649.9(12)	23.8(6)
O3A	6078.3(18)	9822.6(12)	4008.8(11)	14.1(5)
O7A	5335.4(19)	12047.1(13)	3502.7(12)	18.6(5)
O9B	1877.0(19)	3922.6(13)	841.4(12)	17.8(5)
O8B	541.8(19)	3033.5(14)	1628.2(12)	21.1(5)
O5A	5550(2)	12365.9(13)	5078.7(12)	20.8(5)

N1E	3977(3)	7339.0(19)	2613.5(18)	34.6(8)
N1D	5963(3)	7978.7(18)	-1739.3(16)	24.4(7)
N1C	2098(2)	6883.3(17)	6758.9(16)	23.7(7)
C2B	732(3)	5729.0(19)	1031.2(17)	16.8(7)
N1A	10654(3)	7446(2)	4550.3(17)	30.2(8)
C5D	6611(3)	8339(2)	-1382(2)	30.2(9)
C5E	4589(4)	7089(2)	3212(2)	35.5(10)
C3A	8456(3)	8392(2)	4651.8(17)	18.1(7)
O2F	9179(3)	8260(2)	1854(2)	69.6(11)
C4A	9371(3)	8689(2)	4490(2)	25.9(8)
C6A	7278(3)	8928.6(19)	4747.7(17)	16.2(7)
C8A	7139(3)	9289.7(19)	4012.3(17)	16.7(7)
C7C	1003(3)	5362(2)	6097(2)	30.1(9)
C6E	5596(4)	6397(3)	3087(3)	45.9(12)
C9A	6436(3)	8462.3(19)	5056.7(18)	17.2(7)
N1B	3033(3)	7563.7(19)	733.6(17)	29.0(7)
C1BA	1340(3)	7240(2)	754.1(19)	23.0(8)
C1B	1277(3)	6008.4(19)	296.2(18)	16.9(7)
C15D	8056(3)	7181(3)	-3445(2)	36.4(10)
C0BA	1915(3)	6546(2)	436.9(17)	18.3(7)
C3C	2260(3)	7627(2)	8504(2)	28.2(9)
C6D	6654(4)	9151(2)	-1647(2)	38.1(11)
C7E	6139(4)	6231(2)	3721(2)	38.9(11)
C6C	1657(3)	5961(2)	6009(2)	28.7(9)
C5A	10430(3)	8206(2)	4453(2)	29.7(9)
C1C	1985(3)	6982(2)	7530.0(19)	24.6(8)
C2C	2570(3)	7527(2)	7708(2)	28.8(9)
C3B	411(3)	6484(2)	-122.3(18)	18.1(7)
C13E	4745(5)	7501(3)	1933(2)	49.2(13)
C9C	1545(4)	7631(2)	6400(2)	35.2(10)
C11D	5663(3)	5913(2)	-1253(2)	28.3(9)
C7A	7207(3)	9527.0(19)	5276.0(18)	17.6(7)
C9E	3060(4)	8049(2)	2870(2)	42.8(12)
C1A	9783(3)	7159(2)	4682(2)	31.4(9)
C2A	8695(3)	7598(2)	4736(2)	24.8(8)
C5C	1517(3)	6271(2)	6722(2)	26.2(8)
C10D	5407(3)	6708(2)	-1616(2)	27.7(9)
C4B	2091(3)	5341.5(19)	-140.2(18)	16.8(7)
C7AA	3067(3)	6374(2)	284.3(18)	21.9(8)
C7D	7211(4)	9475(3)	-1176(3)	42.8(11)
C10E	2267(4)	8397(3)	2384(2)	43.8(12)
C2E	2692(4)	6452(3)	3030(2)	36.6(10)
C1D	4786(3)	8489(2)	-1707.5(19)	25.9(8)
C2D	4096(3)	8695(2)	-978(2)	33.9(10)
C1E	3525(4)	6714(2)	2446(2)	32.3(9)
C3D	3021(4)	9310(3)	-1029(2)	39.1(10)
C13D	6492(3)	7874(2)	-2510.5(19)	28.3(9)
C9D	5958(3)	7215(2)	-1347(2)	26.3(8)
C2AA	3578(3)	6893(2)	441(2)	27.3(9)
C2F	9511(5)	7311(4)	2677(3)	75.9(19)
C5B	1927(3)	7719(2)	882(2)	27.4(9)

C3E	2273(4)	5866(3)	2739(2)	38.1(10)
C12D	5031(3)	5406(2)	-1447(2)	34.4(10)
C14C	3968(6)	5855(5)	6731(5)	35.1(18)
C12E	570(5)	9486(3)	2249(3)	62.6(15)
C1F	8709(4)	7736(3)	2235(3)	61.7(15)
C11E	1455(5)	9158(3)	2678(3)	59.5(15)
C8C	1224(4)	4935(3)	5427(2)	38.1(10)
C10C	402(4)	8062(2)	6798(2)	37.8(10)
C14D	7665(3)	7362(3)	-2664(2)	39.0(11)
C4D	2190(4)	9409(3)	-323(3)	60.7(15)
C14E	5111(9)	8218(6)	2003(5)	38(2)
C4E	1409(4)	5580(3)	3270(3)	54.5(14)
C3F	8494(5)	8831(4)	1498(3)	79(2)
C4F	8530(6)	8569(4)	780(4)	94(2)
C11C	-277(4)	8657(3)	6340(3)	46.0(12)
C16E	5265(4)	8642(3)	716(2)	47.4(12)
C4C	1089(3)	8126(2)	8767(2)	32.4(9)
C8E	6783(4)	6788(2)	3770(3)	40.8(11)
C16D	7488(4)	6650(3)	-3665(3)	44.0(11)
C8D	8378(4)	9028(3)	-1189(3)	44.6(12)
C12C	300(5)	9248(3)	5997(3)	55.3(13)
C18C	5156(14)	5844(10)	5920(9)	32(4)
C15E	5890(8)	8344(5)	1314(4)	46(3)
C15C	5191(6)	5694(5)	6410(6)	59(3)
C18E	5171(11)	8747(7)	1488(6)	27(4)
C17E	5603(12)	7989(8)	1853(8)	22(3)
C19C	5966(12)	5151(9)	6103(9)	48(6)
C16C	5459(9)	5524(8)	5647(6)	109(5)
C17C	4033(15)	5995(10)	6489(9)	23(4)
C13C	3303(3)	6638(3)	6379(2)	38.3(10)
O1F	2991(5)	6752(4)	4678(3)	46.4(16)

Table S2: Anisotropic Displacement Parameters (Å^2×10^3) for **1**. The Anisotropic displacement factor exponent takes the form: $-2\pi^2[h^2a^{*2}U_{11}+2hka^*b^*U_{12}+\ldots]$.

Atom	U_{11}	U_{22}	U_{33}	U_{23}	U_{13}	U_{12}
Mo1B	13.90(15)	19.24(16)	14.24(15)	-1.91(12)	-2.45(11)	-4.43(12)
Mo3B	13.46(15)	17.08(15)	16.74(15)	-1.50(12)	-5.33(12)	-1.82(12)
Mo1A	15.51(15)	14.39(15)	14.98(15)	-0.93(11)	-3.88(11)	-6.99(12)
Mo2A	16.98(15)	14.56(15)	12.78(14)	1.74(11)	-3.88(11)	-6.80(12)
Mo2B	15.05(15)	17.80(16)	14.87(15)	0.74(11)	-5.13(11)	-4.69(12)
Mo3A	20.38(16)	16.32(15)	12.10(14)	-0.09(11)	-5.55(12)	-7.77(12)
Mn1B	10.2(3)	14.0(4)	13.5(3)	-0.2(3)	-3.4(3)	-3.4(3)
Mn1A	11.1(3)	11.2(3)	11.5(3)	0.2(3)	-3.9(3)	-4.3(3)
O2B	15.8(12)	15.7(12)	15.5(12)	-1.3(9)	-5.3(9)	-5.8(10)
O1B	14.2(12)	15.7(12)	16.7(12)	0.7(9)	-4.3(9)	-6.2(10)
O4B	22.2(13)	26.7(14)	16.8(12)	-3.0(10)	-4.6(10)	-6.7(11)

O6A	13.6(12)	17.0(12)	17.0(12)	-0.4(9)	-2.4(9)	-6.3(10)
O7B	20.0(13)	26.0(14)	19.0(13)	-0.6(10)	-6.5(10)	-5.3(11)
O2A	13.0(11)	12.5(12)	15.2(12)	-1.2(9)	-3.8(9)	-2.0(9)
O6B	16.7(12)	19.3(13)	16.7(12)	1.0(10)	-3.4(10)	-6.1(10)
O4A	21.5(13)	28.9(14)	19.5(13)	-3.6(11)	-4.8(10)	-12.5(11)
O3B	11.6(11)	17.9(12)	17.0(12)	-3.3(9)	-4.8(9)	-5.7(9)
O11B	15.6(12)	17.7(12)	19.1(12)	-2.7(10)	-5.0(10)	-1.9(10)
O10B	17.7(13)	28.4(14)	21.4(13)	-4.7(11)	-5.8(10)	-2.3(11)
O11A	27.7(14)	21.9(13)	24.2(14)	0.8(11)	-14.4(11)	-8.9(11)
O12A	19.0(12)	15.7(12)	15.6(12)	-2.7(9)	-3.3(10)	-7.1(10)
O12B	25.0(14)	19.7(13)	23.3(13)	1.2(10)	-8.4(11)	-2.3(11)
O9A	20.1(12)	18.1(12)	14.2(12)	1.9(9)	-7.0(10)	-7.0(10)
O1A	13.4(11)	13.3(12)	16.5(12)	-0.9(9)	-5.0(9)	-2.0(9)
O5B	17.7(13)	26.0(14)	24.6(14)	-2.8(11)	-1.8(10)	-6.4(11)
O8A	24.5(13)	22.4(13)	17.2(12)	0.7(10)	-1.6(10)	-9.9(11)
O10A	31.5(15)	23.8(14)	17.4(13)	-2.5(10)	-0.6(11)	-13.0(12)
O3A	12.4(11)	14.9(12)	14.4(11)	0.1(9)	-3.4(9)	-2.7(9)
O7A	22.4(13)	17.3(13)	18.3(12)	2.9(10)	-7.7(10)	-7.5(10)
O9B	17.0(12)	21.8(13)	16.7(12)	-1.4(10)	-6.7(10)	-6.0(10)
O8B	20.5(13)	20.4(13)	23.2(13)	2.2(10)	-6.7(10)	-6.3(11)
O5A	24.1(13)	16.8(13)	20.7(13)	-0.5(10)	-2.4(10)	-6.3(10)
N1E	48(2)	28.4(19)	35(2)	-7.2(15)	-13.9(17)	-15.5(17)
N1D	26.2(17)	26.4(17)	23.7(17)	6.6(13)	-12.3(14)	-8.8(14)
N1C	22.8(16)	24.1(16)	24.6(16)	-0.9(12)	-0.3(13)	-11.1(13)
C2B	17.3(17)	18.2(18)	18.5(17)	-2.8(14)	-6.1(14)	-7.7(14)
N1A	19.9(17)	35(2)	30.2(18)	-5.8(15)	-5.2(14)	2.9(15)
C5D	33(2)	33(2)	32(2)	5.9(18)	-18.1(18)	-13.5(18)
C5E	45(3)	30(2)	37(2)	-8.1(18)	-15(2)	-11(2)
C3A	17.1(17)	22.3(19)	15.0(17)	-3.7(14)	-4.9(14)	-3.5(15)
O2F	52(2)	67(3)	79(3)	5(2)	-21(2)	3(2)
C4A	19.6(19)	22(2)	35(2)	-3.2(16)	-5.7(16)	-3.8(16)
C6A	15.0(17)	14.2(17)	19.1(17)	-1.3(13)	-3.3(14)	-3.7(14)
C8A	14.2(17)	15.8(17)	18.3(17)	-1.2(14)	-3.8(14)	-0.9(14)
C7C	28(2)	32(2)	34(2)	-4.3(17)	-9.3(17)	-10.5(17)
C6E	48(3)	36(3)	55(3)	-21(2)	-13(2)	-5(2)
C9A	17.4(17)	14.3(17)	18.0(17)	1.5(14)	-4.7(14)	-1.3(14)
N1B	28.8(18)	33.3(19)	31.5(19)	-5.8(15)	-9.1(15)	-15.1(15)
C1BA	18.7(18)	26(2)	29(2)	-0.9(16)	-10.6(16)	-8.2(16)
C1B	15.9(17)	17.1(18)	18.8(17)	-0.2(14)	-3.9(14)	-6.3(14)
C15D	22(2)	42(3)	38(2)	6(2)	-7.4(18)	0.4(19)
C0BA	23.1(19)	19.8(18)	15.7(17)	-0.4(14)	-7.2(14)	-9.5(15)
C3C	26(2)	29(2)	34(2)	-3.9(17)	-6.7(16)	-14.0(17)
C6D	40(3)	30(2)	51(3)	13(2)	-25(2)	-12(2)
C7E	35(2)	30(2)	50(3)	-8(2)	-12(2)	-1.5(19)
C6C	30(2)	30(2)	29(2)	3.1(16)	-10.9(17)	-10.5(17)
C5A	21(2)	35(2)	33(2)	-8.7(18)	-4.4(17)	-6.8(17)
C1C	24.3(19)	27(2)	25.0(19)	2.3(15)	-5.8(15)	-11.2(16)
C2C	28(2)	25(2)	38(2)	-2.5(17)	-5.9(17)	-14.8(17)
C3B	19.6(18)	19.1(18)	21.2(18)	-0.7(14)	-8.7(14)	-10.6(15)
C13E	76(4)	54(3)	37(3)	2(2)	-19(3)	-45(3)
C9C	56(3)	24(2)	26(2)	5.7(16)	-8.9(19)	-14.3(18)

C11D	28(2)	28(2)	30(2)	-0.6(17)	-9.5(17)	-6.0(17)
C7A	16.1(17)	17.9(18)	19.1(18)	-1.9(14)	-4.3(14)	-4.1(14)
C9E	64(3)	28(2)	45(3)	-15(2)	-28(2)	-6(2)
C1A	29(2)	24(2)	37(2)	-4.2(17)	-7.2(18)	1.2(17)
C2A	22.0(19)	19.8(19)	30(2)	-1.1(16)	-3.8(16)	-3.5(15)
C5C	23.7(19)	26(2)	31(2)	2.6(16)	-6.5(16)	-11.1(16)
C10D	26(2)	28(2)	31(2)	1.0(17)	-11.1(17)	-6.7(17)
C4B	14.1(17)	17.8(18)	20.3(18)	-0.8(14)	-4.1(14)	-6.9(14)
C7AA	20.9(19)	24(2)	22.9(19)	-7.0(15)	-4.8(15)	-7.4(15)
C7D	38(3)	32(2)	60(3)	-3(2)	-12(2)	-11(2)
C10E	59(3)	38(3)	46(3)	0(2)	-25(2)	-21(2)
C2E	41(3)	40(3)	31(2)	-12.1(19)	1.2(19)	-17(2)
C1D	28(2)	27(2)	25(2)	5.1(16)	-11.4(16)	-9.0(17)
C2D	38(2)	42(3)	26(2)	0.3(18)	-11.8(18)	-14(2)
C1E	41(2)	30(2)	31(2)	-10.6(18)	-6.6(19)	-13.9(19)
C3D	40(3)	35(3)	41(3)	-11(2)	-7(2)	-7(2)
C13D	22(2)	37(2)	24(2)	6.4(17)	-10.3(16)	-3.5(17)
C9D	28(2)	29(2)	26(2)	9.5(16)	-11.6(17)	-13.1(17)
C2AA	22(2)	37(2)	27(2)	-4.7(17)	-6.2(16)	-12.7(18)
C2F	43(3)	119(6)	49(3)	4(3)	-4(3)	-4(3)
C5B	32(2)	24(2)	31(2)	-5.3(16)	-11.1(17)	-10.3(17)
C3E	39(3)	42(3)	35(2)	-10(2)	0(2)	-16(2)
C12D	32(2)	35(2)	38(2)	-5.0(19)	-5.3(19)	-11.9(19)
C14C	28(3)	42(4)	35(4)	-9(3)	-7(3)	-8(3)
C12E	72(4)	50(3)	71(4)	7(3)	-34(3)	-13(3)
C1F	42(3)	72(4)	64(4)	15(3)	-5(3)	-15(3)
C11E	87(4)	32(3)	69(4)	2(2)	-47(3)	-7(3)
C8C	40(3)	41(3)	40(2)	-0.9(19)	-16(2)	-16(2)
C10C	38(2)	28(2)	49(3)	2.6(19)	-18(2)	-6.4(18)
C14D	23(2)	56(3)	31(2)	5(2)	-11.3(18)	1(2)
C4D	53(3)	71(4)	50(3)	-17(3)	-6(3)	-3(3)
C14E	27(5)	42(6)	46(5)	-17(4)	1(5)	-14(4)
C4E	55(3)	65(4)	47(3)	-9(3)	5(2)	-31(3)
C3F	67(4)	74(4)	84(5)	-14(4)	-29(4)	14(3)
C4F	86(5)	84(5)	109(6)	-42(4)	-49(4)	19(4)
C11C	50(3)	48(3)	44(3)	7(2)	-17(2)	-16(2)
C16E	57(3)	54(3)	39(3)	-6(2)	-7(2)	-28(3)
C4C	30(2)	32(2)	36(2)	-6.1(18)	-3.7(18)	-10.2(18)
C8E	37(3)	34(2)	53(3)	-18(2)	-10(2)	-5(2)
C16D	42(3)	36(3)	50(3)	1(2)	-6(2)	-9(2)
C8D	49(3)	34(3)	58(3)	4(2)	-24(2)	-15(2)
C12C	66(4)	51(3)	58(3)	0(2)	-21(3)	-24(3)
C18C	42(8)	33(8)	18(8)	-10(6)	0(7)	-7(6)
C15E	47(6)	47(5)	45(5)	-22(4)	11(4)	-25(5)
C15C	27(4)	54(5)	91(7)	-12(4)	4(4)	-13(3)
C18E	26(7)	26(7)	30(7)	-5(5)	-12(5)	-4(6)
C17E	15(7)	16(7)	40(9)	-3(6)	-13(7)	-6(6)
C19C	25(8)	46(9)	60(11)	12(7)	8(7)	-10(6)
C16C	82(8)	113(10)	75(7)	6(6)	22(5)	26(7)
C13C	28(2)	43(3)	45(3)	-15(2)	8.2(18)	-19.5(19)
O1F	39(4)	51(4)	50(4)	-2(3)	-13(3)	-11(3)

Table S3: Bond Lengths for **1**.

Atom	Atom	Length/Å	Atom	Atom	Length/Å
Mo1B	O1B	2.322(2)	N1C	C1C	1.509(5)
Mo1B	O4B	1.705(2)	N1C	C9C	1.527(5)
Mo1B	O6B	1.927(2)	N1C	C5C	1.528(4)
Mo1B	O3B[1]	2.424(2)	N1C	C13C	1.514(5)
Mo1B	O11B[1]	1.922(2)	C2B	C1B	1.549(5)
Mo1B	O5B	1.707(2)	N1A	C5A	1.331(5)
Mo3B	O2B[1]	2.371(2)	N1A	C1A	1.333(5)
Mo3B	O3B	2.340(2)	C5D	C6D	1.525(5)
Mo3B	O11B	1.921(2)	C5E	C6E	1.517(6)
Mo3B	O10B	1.702(2)	C3A	C4A	1.393(5)
Mo3B	O12B	1.697(2)	C3A	C6A	1.532(5)
Mo3B	O9B	1.935(2)	C3A	C2A	1.388(5)
Mo1A	O6A	1.923(2)	O2F	C1F	1.343(6)
Mo1A	O2A[2]	2.375(2)	O2F	C3F	1.399(7)
Mo1A	O4A	1.703(2)	C4A	C5A	1.386(5)
Mo1A	O12A[2]	1.922(2)	C6A	C8A	1.548(4)
Mo1A	O1A	2.350(2)	C6A	C9A	1.543(5)
Mo1A	O5A	1.704(2)	C6A	C7A	1.551(5)
Mo2A	O6A	1.920(2)	C7C	C6C	1.535(5)
Mo2A	O2A[2]	2.450(2)	C7C	C8C	1.523(5)
Mo2A	O9A	1.925(2)	C6E	C7E	1.531(6)
Mo2A	O8A	1.694(2)	N1B	C2AA	1.333(5)
Mo2A	O3A	2.296(2)	N1B	C5B	1.338(5)
Mo2A	O7A	1.716(2)	C1BA	C0BA	1.392(5)
Mo2B	O2B[1]	2.431(2)	C1BA	C5B	1.384(5)
Mo2B	O1B	2.289(2)	C1B	C0BA	1.530(5)
Mo2B	O7B	1.699(2)	C1B	C3B	1.548(5)
Mo2B	O6B	1.923(2)	C1B	C4B	1.538(5)
Mo2B	O9B	1.914(2)	C15D	C14D	1.534(6)
Mo2B	O8B	1.711(2)	C15D	C16D	1.519(6)
Mo3A	O11A	1.707(2)	C0BA	C7AA	1.393(5)
Mo3A	O12A	1.929(2)	C3C	C2C	1.534(5)
Mo3A	O9A	1.924(2)	C3C	C4C	1.520(5)
Mo3A	O1A[2]	2.400(2)	C6D	C7D	1.547(6)
Mo3A	O10A	1.697(2)	C7E	C8E	1.508(6)
Mo3A	O3A	2.326(2)	C6C	C5C	1.515(5)
Mn1B	O2B	1.913(2)	C1C	C2C	1.519(5)
Mn1B	O2B[1]	1.913(2)	C13E	C14E	1.542(10)
Mn1B	O1B	2.102(2)	C13E	C17E	1.581(14)
Mn1B	O1B[1]	2.102(2)	C9C	C10C	1.522(6)
Mn1B	O3B[1]	1.927(2)	C11D	C10D	1.524(5)
Mn1B	O3B	1.927(2)	C11D	C12D	1.523(5)
Mn1A	O2A	1.915(2)	C9E	C10E	1.523(6)
Mn1A	O2A[2]	1.915(2)	C1A	C2A	1.385(5)
Mn1A	O1A[2]	1.939(2)	C10D	C9D	1.516(5)
Mn1A	O1A	1.939(2)	C7AA	C2AA	1.392(5)

Mn1A	O3A[2]	2.099(2)	C7D	C8D	1.486(6)
Mn1A	O3A	2.099(2)	C10E	C11E	1.546(7)
O2B	Mo3B[1]	2.371(2)	C2E	C1E	1.514(5)
O2B	Mo2B[1]	2.431(2)	C2E	C3E	1.537(6)
O2B	C3B	1.439(4)	C1D	C2D	1.514(5)
O1B	C2B	1.432(4)	C2D	C3D	1.527(6)
O2A	Mo1A[2]	2.375(2)	C3D	C4D	1.529(6)
O2A	Mo2A[2]	2.450(2)	C13D	C14D	1.510(5)
O2A	C9A	1.441(4)	C2F	C1F	1.498(7)
O3B	Mo1B[1]	2.424(2)	C3E	C4E	1.507(6)
O3B	C4B	1.437(4)	C14C	C15C	1.507(10)
O11B	Mo1B[1]	1.921(2)	C14C	C13C	1.614(10)
O12A	Mo1A[2]	1.922(2)	C12E	C11E	1.524(7)
O1A	Mo3A[2]	2.400(2)	C10C	C11C	1.536(6)
O1A	C7A	1.440(4)	C14E	C15E	1.520(13)
O3A	C8A	1.432(4)	C3F	C4F	1.518(8)
N1E	C5E	1.524(5)	C11C	C12C	1.496(6)
N1E	C13E	1.519(6)	C16E	C15E	1.543(10)
N1E	C9E	1.514(5)	C16E	C18E	1.517(12)
N1E	C1E	1.516(5)	C18C	C19C	1.46(3)
N1D	C5D	1.521(5)	C18C	C17C	1.58(2)
N1D	C1D	1.525(5)	C15C	C16C	1.499(17)
N1D	C13D	1.511(5)	C18E	C17E	1.503(19)
N1D	C9D	1.516(4)	C17C	C13C	1.307(18)

[1]-X,1-Y,-Z; [2]1-X,2-Y,1-Z

Table S4: Bond Angles for **1**.

Atom	Atom	Atom	Angle/˚	Atom	Atom	Atom	Angle/˚
O1B	Mo1B	O3B[1]	71.92(7)	C2B	O1B	Mo2B	122.35(19)
O4B	Mo1B	O1B	94.11(10)	C2B	O1B	Mn1B	114.25(18)
O4B	Mo1B	O6B	102.74(11)	Mo2A	O6A	Mo1A	123.01(11)
O4B	Mo1B	O3B[1]	164.01(10)	Mo1A[2]	O2A	Mo2A[2]	88.85(7)
O4B	Mo1B	O11B[1]	100.33(11)	Mn1A	O2A	Mo1A[2]	102.10(9)
O4B	Mo1B	O5B	105.84(12)	Mn1A	O2A	Mo2A[2]	99.91(9)
O6B	Mo1B	O1B	70.74(9)	C9A	O2A	Mo1A[2]	120.28(18)
O6B	Mo1B	O3B[1]	80.40(9)	C9A	O2A	Mo2A[2]	122.51(18)
O11B[1]	Mo1B	O1B	81.19(9)	C9A	O2A	Mn1A	117.67(18)
O11B[1]	Mo1B	O6B	144.60(10)	Mo2B	O6B	Mo1B	120.16(12)
O11B[1]	Mo1B	O3B[1]	70.47(9)	Mo3B	O3B	Mo1B[1]	89.50(7)
O5B	Mo1B	O1B	158.93(10)	Mn1B	O3B	Mo1B[1]	101.22(9)
O5B	Mo1B	O6B	97.73(11)	Mn1B	O3B	Mo3B	102.45(9)
O5B	Mo1B	O3B[1]	89.08(10)	C4B	O3B	Mo1B[1]	122.31(18)
O5B	Mo1B	O11B[1]	101.37(11)	C4B	O3B	Mo3B	118.80(18)
O3B	Mo3B	O2B[1]	68.48(7)	C4B	O3B	Mn1B	117.51(19)
O11B	Mo3B	O2B[1]	80.42(9)	Mo3B	O11B	Mo1B[1]	121.57(12)
O11B	Mo3B	O3B	72.42(9)	Mo1A[2]	O12A	Mo3A	121.75(12)
O11B	Mo3B	O9B	145.36(10)	Mo3A	O9A	Mo2A	120.15(12)

O10B	Mo3B	O2B^{1}	161.55(10)	Mo1A	O1A	Mo3A^{2}	90.17(8)
O10B	Mo3B	O3B	94.76(10)	Mn1A	O1A	Mo1A	102.27(9)
O10B	Mo3B	O11B	102.44(11)	Mn1A	O1A	Mo3A^{2}	101.33(9)
O10B	Mo3B	O9B	98.33(10)	C7A	O1A	Mo1A	119.82(18)
O12B	Mo3B	O2B^{1}	91.38(10)	C7A	O1A	Mo3A^{2}	121.42(18)
O12B	Mo3B	O3B	159.07(10)	C7A	O1A	Mn1A	117.00(19)
O12B	Mo3B	O11B	99.48(11)	Mo2A	O3A	Mo3A	92.36(8)
O12B	Mo3B	O10B	105.93(12)	Mn1A	O3A	Mo2A	99.59(9)
O12B	Mo3B	O9B	101.08(11)	Mn1A	O3A	Mo3A	99.01(8)
O9B	Mo3B	O2B^{1}	71.53(8)	C8A	O3A	Mo2A	121.91(18)
O9B	Mo3B	O3B	78.53(9)	C8A	O3A	Mo3A	123.98(19)
O6A	Mo1A	O2A^{2}	71.96(8)	C8A	O3A	Mn1A	114.74(18)
O6A	Mo1A	O1A	79.98(9)	Mo2B	O9B	Mo3B	122.54(12)
O4A	Mo1A	O6A	98.94(10)	C13E	N1E	C5E	111.6(3)
O4A	Mo1A	O2A^{2}	160.23(10)	C9E	N1E	C5E	104.6(3)
O4A	Mo1A	O12A^{2}	101.91(10)	C9E	N1E	C13E	112.2(4)
O4A	Mo1A	O1A	93.00(10)	C9E	N1E	C1E	111.9(3)
O4A	Mo1A	O5A	105.68(12)	C1E	N1E	C5E	111.9(3)
O12A^{2}	Mo1A	O6A	145.58(9)	C1E	N1E	C13E	104.9(3)
O12A^{2}	Mo1A	O2A^{2}	79.39(8)	C5D	N1D	C1D	111.4(3)
O12A^{2}	Mo1A	O1A	71.90(9)	C13D	N1D	C5D	111.3(3)
O1A	Mo1A	O2A^{2}	68.44(7)	C13D	N1D	C1D	106.0(3)
O5A	Mo1A	O6A	101.44(10)	C13D	N1D	C9D	111.6(3)
O5A	Mo1A	O2A^{2}	93.48(10)	C9D	N1D	C5D	105.4(3)
O5A	Mo1A	O12A^{2}	98.91(10)	C9D	N1D	C1D	111.2(3)
O5A	Mo1A	O1A	160.68(10)	C1C	N1C	C9C	111.3(3)
O6A	Mo2A	O2A^{2}	70.25(8)	C1C	N1C	C5C	106.1(3)
O6A	Mo2A	O9A	144.48(9)	C1C	N1C	C13C	111.4(3)
O6A	Mo2A	O3A	81.54(9)	C9C	N1C	C5C	109.7(3)
O9A	Mo2A	O2A^{2}	79.55(8)	C13C	N1C	C9C	107.6(3)
O9A	Mo2A	O3A	71.55(8)	C13C	N1C	C5C	110.9(3)
O8A	Mo2A	O6A	100.49(11)	O1B	C2B	C1B	114.3(3)
O8A	Mo2A	O2A^{2}	163.23(10)	C5A	N1A	C1A	115.8(3)
O8A	Mo2A	O9A	103.57(11)	N1D	C5D	C6D	115.6(3)
O8A	Mo2A	O3A	92.96(10)	C6E	C5E	N1E	116.3(3)
O8A	Mo2A	O7A	105.39(11)	C4A	C3A	C6A	120.7(3)
O3A	Mo2A	O2A^{2}	72.20(7)	C2A	C3A	C4A	115.5(3)
O7A	Mo2A	O6A	101.06(10)	C2A	C3A	C6A	123.8(3)
O7A	Mo2A	O2A^{2}	90.36(9)	C1F	O2F	C3F	115.8(5)
O7A	Mo2A	O9A	97.31(10)	C5A	C4A	C3A	120.5(4)
O7A	Mo2A	O3A	160.52(10)	C3A	C6A	C8A	106.7(3)
O1B	Mo2B	O2B^{1}	72.32(8)	C3A	C6A	C9A	109.4(3)
O7B	Mo2B	O2B^{1}	163.00(10)	C3A	C6A	C7A	105.7(3)
O7B	Mo2B	O1B	92.47(10)	C8A	C6A	C7A	113.1(3)
O7B	Mo2B	O6B	103.57(11)	C9A	C6A	C8A	111.6(3)
O7B	Mo2B	O9B	100.56(10)	C9A	C6A	C7A	110.1(3)
O7B	Mo2B	O8B	105.38(11)	O3A	C8A	C6A	114.0(3)
O6B	Mo2B	O2B^{1}	79.25(9)	C8C	C7C	C6C	112.5(3)
O6B	Mo2B	O1B	71.56(9)	C5E	C6E	C7E	110.3(4)
O9B	Mo2B	O2B^{1}	70.47(8)	O2A	C9A	C6A	114.4(3)
O9B	Mo2B	O1B	82.14(9)	C2AA	N1B	C5B	115.5(3)

O9B	Mo2B	O6B	144.73(10)	C5B	C1BA	C0BA	119.3(3)
O8B	Mo2B	O2B^{1}	90.71(10)	C0BA	C1B	C2B	105.4(3)
O8B	Mo2B	O1B	160.97(10)	C0BA	C1B	C3B	107.3(3)
O8B	Mo2B	O6B	97.36(10)	C0BA	C1B	C4B	109.1(3)
O8B	Mo2B	O9B	100.59(11)	C3B	C1B	C2B	112.4(3)
O11A	Mo3A	O12A	100.79(10)	C4B	C1B	C2B	112.2(3)
O11A	Mo3A	O9A	97.61(11)	C4B	C1B	C3B	110.1(3)
O11A	Mo3A	O1A^{2}	90.78(10)	C16D	C15D	C14D	113.4(4)
O11A	Mo3A	O3A	160.78(10)	C1BA	C0BA	C1B	119.9(3)
O12A	Mo3A	O1A^{2}	70.62(8)	C1BA	C0BA	C7AA	116.4(3)
O12A	Mo3A	O3A	82.57(8)	C7AA	C0BA	C1B	123.7(3)
O9A	Mo3A	O12A	145.00(9)	C4C	C3C	C2C	113.8(3)
O9A	Mo3A	O1A^{2}	79.66(9)	C5D	C6D	C7D	109.4(3)
O9A	Mo3A	O3A	70.87(8)	C8E	C7E	C6E	113.5(4)
O10A	Mo3A	O11A	105.73(12)	C5C	C6C	C7C	108.6(3)
O10A	Mo3A	O12A	100.90(11)	N1A	C5A	C4A	123.8(4)
O10A	Mo3A	O9A	102.39(11)	N1C	C1C	C2C	116.3(3)
O10A	Mo3A	O1A^{2}	162.80(10)	C1C	C2C	C3C	109.1(3)
O10A	Mo3A	O3A	92.03(10)	O2B	C3B	C1B	114.4(3)
O3A	Mo3A	O1A^{2}	72.34(7)	N1E	C13E	C14E	109.6(5)
O2B	Mn1B	O2B^{1}	180.0	N1E	C13E	C17E	125.6(7)
O2B^{1}	Mn1B	O1B^{1}	92.20(9)	C10C	C9C	N1C	115.4(3)
O2B	Mn1B	O1B	92.21(9)	C12D	C11D	C10D	112.0(3)
O2B	Mn1B	O1B^{1}	87.79(9)	O1A	C7A	C6A	114.5(3)
O2B^{1}	Mn1B	O1B	87.80(9)	N1E	C9E	C10E	115.7(3)
O2B	Mn1B	O3B^{1}	87.32(9)	N1A	C1A	C2A	124.4(4)
O2B^{1}	Mn1B	O3B^{1}	92.68(9)	C1A	C2A	C3A	120.0(4)
O2B^{1}	Mn1B	O3B	87.32(9)	C6C	C5C	N1C	117.6(3)
O2B	Mn1B	O3B	92.68(9)	C9D	C10D	C11D	109.9(3)
O1B^{1}	Mn1B	O1B	180.0	O3B	C4B	C1B	114.3(3)
O3B^{1}	Mn1B	O1B	87.48(9)	C2AA	C7AA	C0BA	119.8(3)
O3B^{1}	Mn1B	O1B^{1}	92.52(9)	C8D	C7D	C6D	113.4(4)
O3B	Mn1B	O1B^{1}	87.48(9)	C9E	C10E	C11E	109.7(4)
O3B	Mn1B	O1B	92.52(9)	C1E	C2E	C3E	108.6(3)
O3B	Mn1B	O3B^{1}	180.0	C2D	C1D	N1D	115.7(3)
O2A	Mn1A	O2A^{2}	180.0	C1D	C2D	C3D	110.0(3)
O2A	Mn1A	O1A^{2}	87.17(9)	C2E	C1E	N1E	116.8(3)
O2A^{2}	Mn1A	O1A^{2}	92.83(9)	C2D	C3D	C4D	111.7(4)
O2A^{2}	Mn1A	O1A	87.17(9)	C14D	C13D	N1D	114.9(3)
O2A	Mn1A	O1A	92.83(9)	C10D	C9D	N1D	117.0(3)
O2A	Mn1A	O3A	91.70(9)	N1B	C2AA	C7AA	124.1(4)
O2A	Mn1A	O3A^{2}	88.30(9)	N1B	C5B	C1BA	124.9(4)
O2A^{2}	Mn1A	O3A	88.30(9)	C4E	C3E	C2E	113.4(4)
O2A^{2}	Mn1A	O3A^{2}	91.70(9)	C15C	C14C	C13C	109.7(7)
O1A	Mn1A	O1A^{2}	180.00(6)	O2F	C1F	C2F	104.9(5)
O1A^{2}	Mn1A	O3A	87.31(9)	C12E	C11E	C10E	111.6(4)
O1A	Mn1A	O3A^{2}	87.31(9)	C9C	C10C	C11C	113.9(4)
O1A	Mn1A	O3A	92.69(9)	C13D	C14D	C15D	110.9(3)
O1A^{2}	Mn1A	O3A^{2}	92.69(9)	C15E	C14E	C13E	107.3(7)
O3A	Mn1A	O3A^{2}	180.00(11)	O2F	C3F	C4F	110.7(5)
Mo3B^{1}	O2B	Mo2B^{1}	89.32(7)	C12C	C11C	C10C	112.5(4)

Mn1B	O2B	Mo3B1	101.75(9)	C19C	C18C	C17C	111.2(14)
Mn1B	O2B	Mo2B1	100.37(9)	C14E	C15E	C16E	111.9(8)
C3B	O2B	Mo3B1	121.06(19)	C16C	C15C	C14C	112.4(8)
C3B	O2B	Mo2B1	121.53(18)	C17E	C18E	C16E	110.6(11)
C3B	O2B	Mn1B	117.45(19)	C18E	C17E	C13E	108.7(10)
Mo2B	O1B	Mo1B	92.71(8)	C13C	C17C	C18C	113.9(12)
Mn1B	O1B	Mo1B	99.39(8)	N1C	C13C	C14C	111.4(4)
Mn1B	O1B	Mo2B	99.47(9)	C17C	C13C	N1C	126.4(8)
C2B	O1B	Mo1B	123.57(19)				

[1]-X,1-Y,-Z; [2]1-X,2-Y,1-Z

Table S5: Hydrogen Atom Coordinates (Å×10^4) and Isotropic Displacement Parameters (Å^2×10^3) for **1**.

**Atom**	***x***	***y***	***z***	**U(eq)**
H2BA	1310	5470	1281	20
H2BB	265	6173	1293	20
H5DA	7365	8011	-1443	36
H5DB	6286	8349	-882	36
H5EA	4822	7519	3312	43
H5EB	4069	6977	3627	43
H4A	9269	9215	4406	31
H8AA	7709	9552	3828	20
H8AB	7253	8881	3699	20
H7CA	1203	4994	6472	36
H7CB	217	5617	6233	36
H6EA	6126	6496	2670	55
H6EB	5374	5953	3008	55
H9AA	6513	8080	4725	21
H9AB	6617	8192	5485	21
H1BA	570	7380	879	28
H15E	7918	7658	-3723	44
H15F	8850	6945	-3545	44
H3CA	2336	7124	8740	34
H3CB	2779	7853	8631	34
H6DA	5909	9478	-1635	46
H6DB	7073	9142	-2129	46
H7EA	5568	6248	4147	47
H7EB	6635	5715	3695	47
H6CA	2436	5726	5816	34
H6CB	1384	6376	5688	34
H5A	11019	8425	4355	36
H1CA	2268	6481	7746	29
H1CB	1203	7163	7744	29
H2CA	2349	8021	7465	35
H2CB	3366	7322	7556	35
H3BA	-55	6924	146	22
H3BB	798	6677	-559	22
H13E	4363	7585	1548	59

H13F	5390	7062	1829	59
H13G	4269	7741	1609	59
H13H	5172	7002	1750	59
H9CA	2026	7968	6324	42
H9CB	1482	7516	5942	42
H11E	6452	5668	-1386	34
H11F	5469	5966	-748	34
H7AB	7367	9257	5709	21
H7AC	7776	9789	5085	21
H9EA	3397	8437	2946	51
H9EB	2633	7920	3321	51
H1A	9914	6629	4741	38
H2A	8126	7361	4829	30
H5CA	1777	5842	7034	31
H5CB	730	6484	6908	31
H10E	5676	6664	-2120	33
H10F	4612	6937	-1523	33
H4BA	2487	5553	-563	20
H4BB	2632	5057	129	20
H7AA	3494	5915	79	26
H7DA	7193	10002	-1330	51
H7DB	6785	9479	-696	51
H10C	1854	8040	2346	53
H10D	2683	8494	1918	53
H2EA	2076	6888	3202	44
H2EB	3038	6217	3418	44
H1DA	4828	8962	-1979	31
H1DB	4404	8232	-1933	31
H2DA	4511	8884	-718	41
H2DB	3925	8241	-728	41
H1EA	3180	6896	2048	39
H1EB	4145	6271	2300	39
H3DA	2693	9169	-1375	47
H3DB	3186	9795	-1188	47
H13I	6039	7660	-2720	34
H13J	6488	8375	-2737	34
H9DA	6720	6927	-1353	32
H9DB	5591	7317	-860	32
H2AA	4349	6762	334	33
H2FA	10080	6917	2408	114
H2FB	9127	7081	3085	114
H2FC	9841	7662	2820	114
H5B	1522	8183	1087	33
H3EA	1963	6102	2338	46
H3EB	2897	5431	2575	46
H12G	5207	4914	-1205	52
H12H	5238	5339	-1945	52
H12I	4249	5645	-1313	52
H14A	3840	5906	7234	42
H14B	3702	5431	6653	42
H12D	920	9546	1764	94

H12E	118	9977	2421	94
H12F	116	9142	2291	94
H1F	8033	7655	2226	74
H11C	1101	9068	3162	71
H11D	1866	9529	2672	71
H8CA	766	4590	5499	57
H8CB	1990	4649	5311	57
H8CC	1054	5298	5050	57
H10A	487	8322	7180	45
H10B	-9	7693	7003	45
H14E	7702	6887	-2382	47
H14F	8152	7616	-2535	47
H4DA	2501	9569	16	91
H4DB	2026	8930	-164	91
H4DC	1520	9791	-376	91
H14C	4472	8662	2095	45
H14D	5489	8140	2389	45
H4EA	1175	5223	3056	82
H4EB	783	6006	3431	82
H4EC	1717	5329	3661	82
H3F	8074	9306	1676	95
H4FA	9244	8543	481	141
H4FB	7967	8929	577	141
H4FC	8400	8071	830	141
H11A	-416	8394	5980	55
H11B	-987	8911	6631	55
H16D	5778	8730	295	71
H16E	4708	9114	846	71
H16F	4920	8267	636	71
H16G	6024	8407	500	71
H16H	5015	9133	487	71
H16I	4815	8319	672	71
H4CA	948	8160	9268	49
H4CB	567	7903	8652	49
H4CC	1012	8631	8549	49
H8EA	7379	6752	3364	61
H8EB	7079	6665	4187	61
H8EC	6300	7301	3791	61
H16J	7544	6202	-3355	66
H16K	7840	6499	-4138	66
H16L	6721	6914	-3643	66
H8DA	8711	9296	-945	67
H8DB	8784	8967	-1668	67
H8DC	8392	8531	-964	67
H12A	523	9465	6345	83
H12B	-196	9646	5775	83
H12C	942	9012	5650	83
H18A	5015	5789	5466	39
H18B	5456	6281	5882	39
H15C	6445	7864	1184	55
H15D	6269	8712	1377	55

H15A	5439	6135	6463	71
H15B	5592	5259	6663	71
H18C	5591	9097	1532	32
H18D	4401	8971	1710	32
H17C	5697	8073	2312	26
H17D	6317	7715	1580	26
H19A	5810	5058	6604	72
H19B	6697	5218	5949	72
H19C	5922	4721	5878	72
H16A	5109	5144	5583	163
H16B	6247	5333	5487	163
H16C	5191	5986	5381	163
H17A	3713	5572	6499	28
H17B	4192	6001	6948	28
H13C	3647	7042	6395	46
H13D	3347	6559	5890	46
H13A	3297	6658	5884	46
H13B	3616	7044	6442	46
H1FA	3167	6840	5047	70
H1FB	2311	6999	4726	70

Table S6: Atomic Occupancy for **1**.

**Atom**	***Occupancy***	**Atom**	***Occupancy***	**Atom**	***Occupancy***
H13E	0.637(14)	H13F	0.637(14)	H13G	0.363(14)
H13H	0.363(14)	C14C	0.693(13)	H14A	0.693(13)
H14B	0.693(13)	C14E	0.637(14)	H14C	0.637(14)
H14D	0.637(14)	H16D	0.637(14)	H16E	0.637(14)
H16F	0.637(14)	H16G	0.363(14)	H16H	0.363(14)
H16I	0.363(14)	C18C	0.307(13)	H18A	0.307(13)
H18B	0.307(13)	C15E	0.637(14)	H15C	0.637(14)
H15D	0.637(14)	C15C	0.693(13)	H15A	0.693(13)
H15B	0.693(13)	C18E	0.363(14)	H18C	0.363(14)
H18D	0.363(14)	C17E	0.363(14)	H17C	0.363(14)
H17D	0.363(14)	C19C	0.307(13)	H19A	0.307(13)
H19B	0.307(13)	H19C	0.307(13)	C16C	0.693(13)
H16A	0.693(13)	H16B	0.693(13)	H16C	0.693(13)
C17C	0.307(13)	H17A	0.307(13)	H17B	0.307(13)
H13C	0.693(13)	H13D	0.693(13)	H13A	0.307(13)
H13B	0.307(13)	O1F	0.5	H1FA	0.5
H1FB	0.5				

Crystal structure determination of **1**

Crystal Data for $C_{70}H_{137}MnMo_6N_5O_{25.5}$ (M =2087.42 g/mol): triclinic, space group P-1 (no. 2), a = 12.9111(12) Å, b = 18.2011(18) Å, c = 19.6030(16) Å, α = 83.907(3)°, β = 76.265(2)°, γ = 73.172(2)°, V = 4279.8(7) Å^3, Z = 2, T = 150 K, μ(MoKα) = 1.067 mm^{-1}, $Dcalc$ = 1.620 g/cm^3, 118631 reflections measured

(3.106° ≤ 2Θ ≤ 52.874°), 17584 unique (R_{int} = 0.0620, R_{sigma} = 0.0392) which were used in all calculations. The final R_1 was 0.0350 (I > 2σ(I)) and wR_2 was 0.0831 (all data).

Refinement model description

Number of restraints - 105, number of constraints - unknown.

Details:

```
1. Fixed Uiso
 At 1.2 times of:
  All C(H) groups, All C(H,H) groups, All C(H,H,H,H) groups
 At 1.5 times of:
  All C(H,H,H) groups, All C(H,H,H,H,H,H) groups, All O(H,H) groups
2. Rigid body (RIGU) restrains
 C13C, N1C, C1C, C9C, C5C, H1CA, H1CB, C2C, H9CA, H9CB, C10C, C6C, H5CA, H5CB,
 C14C, C17C, C3C, H2CA, H2CB, H10A, H10B, C11C, C7C, H6CA, H6CB, H14A, H14B,
 C15C, C18C, H3CA, H3CB, C4C, H11A, H11B, C12C, H7CA, H7CB, C8C, H15A, H15B,
 C16C, H18A, H18B, C19C, H4CA, H4CB, H4CC, H12A, H12B, H12C, H8CA, H8CB, H8CC,
 H16A, H16B, H16C, H19A, H19B, H19C
 with sigma for 1-2 distances of 0.004 and sigma for 1-3 distances of 0.004
3. Others
 Sof(H13G)=Sof(H13H)=Sof(H16G)=Sof(H16H)=Sof(H16I)=Sof(C18E)=Sof(H18C)=
 Sof(H18D)=Sof(C17E)=Sof(H17C)=Sof(H17D)=1-FVAR(1)
 Sof(H13E)=Sof(H13F)=Sof(C14E)=Sof(H14C)=Sof(H14D)=Sof(H16D)=Sof(H16E)=
 Sof(H16F)=Sof(C15E)=Sof(H15C)=Sof(H15D)=FVAR(1)
 Sof(C18C)=Sof(H18A)=Sof(H18B)=Sof(C19C)=Sof(H19A)=Sof(H19B)=Sof(H19C)=
 Sof(C17C)=Sof(H17A)=Sof(H17B)=Sof(H13A)=Sof(H13B)=1-FVAR(2)
 Sof(C14C)=Sof(H14A)=Sof(H14B)=Sof(C15C)=Sof(H15A)=Sof(H15B)=Sof(C16C)=
 Sof(H16A)=Sof(H16B)=Sof(H16C)=Sof(H13C)=Sof(H13D)=FVAR(2)
 Fixed Sof: O1F(0.5) H1FA(0.5) H1FB(0.5)
4.a Free rotating group:
 O1F(H1FA,H1FB)
4.b Secondary CH2 refined with riding coordinates:
 C2B(H2BA,H2BB), C5D(H5DA,H5DB), C5E(H5EA,H5EB), C8A(H8AA,H8AB), C7C(H7CA,
 H7CB), C6E(H6EA,H6EB), C9A(H9AA,H9AB), C15D(H15E,H15F), C3C(H3CA,H3CB),
 C6D(H6DA,H6DB), C7E(H7EA,H7EB), C6C(H6CA,H6CB), C1C(H1CA,H1CB), C2C(H2CA,H2CB),
  C3B(H3BA,H3BB), C13E(H13E,H13F), C13E(H13G,H13H), C9C(H9CA,H9CB), C11D(H11E,
 H11F), C7A(H7AB,H7AC), C9E(H9EA,H9EB), C5C(H5CA,H5CB), C10D(H10E,H10F),
 C4B(H4BA,H4BB), C7D(H7DA,H7DB), C10E(H10C,H10D), C2E(H2EA,H2EB), C1D(H1DA,
 H1DB), C2D(H2DA,H2DB), C1E(H1EA,H1EB), C3D(H3DA,H3DB), C13D(H13I,H13J),
 C9D(H9DA,H9DB), C3E(H3EA,H3EB), C14C(H14A,H14B), C11E(H11C,H11D), C10C(H10A,
 H10B), C14D(H14E,H14F), C14E(H14C,H14D), C11C(H11A,H11B), C18C(H18A,H18B),
 C15E(H15C,H15D), C15C(H15A,H15B), C18E(H18C,H18D), C17E(H17C,H17D), C17C(H17A,
 H17B), C13C(H13C,H13D), C13C(H13A,H13B)
4.c Aromatic/amide H refined with riding coordinates:
 C4A(H4A), C1BA(H1BA), C5A(H5A), C1A(H1A), C2A(H2A), C7AA(H7AA), C2AA(H2AA),
 C5B(H5B), C1F(H1F), C3F(H3F)
4.d Idealised Me refined as rotating group:
 C2F(H2FA,H2FB,H2FC), C12D(H12G,H12H,H12I), C12E(H12D,H12E,H12F), C8C(H8CA,
 H8CB,H8CC), C4D(H4DA,H4DB,H4DC), C4E(H4EA,H4EB,H4EC), C4F(H4FA,H4FB,H4FC),
 C16E(H16D,H16E,H16F), C16E(H16G,H16H,H16I), C4C(H4CA,H4CB,H4CC), C8E(H8EA,H8EB,
 H8EC), C16D(H16J,H16K,H16L), C8D(H8DA,H8DB,H8DC), C12C(H12A,H12B,H12C),
 C19C(H19A,H19B,H19C), C16C(H16A,H16B,H16C)
```

7. References

(1) Yazigi, F. J.; Wilson, C.; Long, D. L.; Forgan, R. S. Synthetic Considerations in the Self-Assembly of Coordination Polymers of Pyridine-Functionalized Hybrid Mn-Anderson Polyoxometalates. *Cryst. Growth Des.* **2017**, *17* (9), 4739–4748. https://doi.org/10.1021/acs.cgd.7b00672.

(2) Xu, B.; Tao, N. Measurement of Single-Molecule Resistance by Repeated Formation of Molecular Junctions. *Science* **2003**, *301* (5637), 1221–1223. https://doi.org/10.1126/science.1087481.

(3) Klemperer, W. G.; Shum, W. Synthesis and Interconversion of the Isomeric .Alpha.- and .Beta.-Molybdate (Mo8O264-) Ions. *J. Am. Chem. Soc.* **1976**, *98* (25), 8291–8293. https://doi.org/10.1021/ja00441a083.

(4) Mészáros, G.; Li, C.; Pobelov, I.; Wandlowski, T. Current Measurements in a Wide Dynamic Range-Applications in Electrochemical Nanotechnology. *Nanotechnology* **2007**, *18* (42), 424004. https://doi.org/10.1088/0957-4484/18/42/424004.

(5) Dolomanov, O. V.; Bourhis, L. J.; Gildea, R. J.; Howard, J. A. K.; Puschmann, H. OLEX2 : A Complete Structure Solution, Refinement and Analysis Program. *J. Appl. Crystallogr.* **2009**, *42* (2), 339–341. https://doi.org/10.1107/S0021889808042726.

(6) Sheldrick, G. M. SHELXT - Integrated Space-Group and Crystal-Structure Determination. *Acta Crystallogr. Sect. A Found. Crystallogr.* **2015**, *71* (1), 3–8. https://doi.org/10.1107/S2053273314026370.

(7) Sheldrick, G. M. Crystal Structure Refinement with SHELXL. *Acta Crystallogr. Sect. C Struct. Chem.* **2015**, *71* (Md), 3–8. https://doi.org/10.1107/S2053229614024218.
